# Thirty-One Novel Biomarkers as Predictors for Clinically Incident Diabetes

**DOI:** 10.1371/journal.pone.0010100

**Published:** 2010-04-09

**Authors:** Veikko Salomaa, Aki Havulinna, Olli Saarela, Tanja Zeller, Pekka Jousilahti, Antti Jula, Thomas Muenzel, Arpo Aromaa, Alun Evans, Kari Kuulasmaa, Stefan Blankenberg

**Affiliations:** 1 Department of Chronic Disease Prevention, National Institute for Health and Welfare, Helsinki, Finland; 2 The UKCRC Centre of Excellence for Public Health, Queen's University of Belfast, Belfast, United Kingdom; 3 Department of Medicine II, Johannes Gutenberg-University, Mainz, Germany; University of Bremen, Germany

## Abstract

**Background:**

The prevalence of diabetes is increasing in all industrialized countries and its prevention has become a public health priority. However, the predictors of diabetes risk are insufficiently understood. We evaluated, whether 31 novel biomarkers could help to predict the risk of incident diabetes.

**Methods and Findings:**

The biomarkers were evaluated primarily in the FINRISK97 cohort (n = 7,827; 417 cases of clinically incident diabetes during the follow-up). The findings were replicated in the Health 2000 cohort (n = 4,977; 179 cases of clinically incident diabetes during the follow-up). We used Cox proportional hazards models to calculate the relative risk of diabetes, after adjusting for the classic risk factors, separately for each biomarker. Next, we assessed the discriminatory ability of single biomarkers using receiver operating characteristic curves and C-statistics, integrated discrimination improvement (IDI) and net reclassification improvement (NRI). Finally, we derived a biomarker score in the FINRISK97 cohort and validated it in the Health 2000 cohort. A score consisting of adiponectin, apolipoprotein B, C-reactive protein and ferritin almost doubled the relative risk of diabetes in the validation cohort (HR per one standard deviation increase 1.88, p = 2.8 e-5). It also improved discrimination of the model (IDI = 0.0149, p<0.0001) and reclassification of diabetes risk (NRI = 11.8%, p = 0.006). Gender-specific analyses suggested that the best score differed between men and women. Among men, the best results were obtained with the score of four biomarkers: adiponectin, apolipoprotein B, ferritin and interleukin-1 receptor antagonist, which gave an NRI of 25.4% (p<0.0001). Among women, the best score included adiponectin, apolipoprotein B, C-reactive protein and insulin. It gave an NRI of 13.6% (p = 0.041).

**Conclusions:**

We identified novel biomarkers that were associated with the risk of clinically incident diabetes over and above the classic risk factors. This gives new insights into the pathogenesis of diabetes and may help with targeting prevention and treatment.

## Introduction

Diabetes and its complications have become a major public health problem in all western countries. It was estimated that 12.9% of the U.S. population aged ≥20 years had diabetes in 2005–2006 and the proportion increased to 31.6% in persons aged ≥65 years [1]. It is well known that diabetes increases the risk of coronary heart disease by 2–3 fold in men and by 3–4 fold in women [2], [3]. It also increases the risk of stroke by 1.5–4 fold and accounts for 35–45% of cases of end-stage renal failure [4], [5]. Recent research has shown that the onset of type 2 diabetes can be postponed or prevented with lifestyle intervention or by medication [6], [7]. Identifying individuals at high risk of diabetes has therefore become a priority for targeting preventive measures effectively.

Several risk equations based on lifestyle factors [8], classic clinical risk factors [9] and genetic factors [10], [11] have been proposed and tested for the prediction of diabetes. The performance of these equations is fairly good but none has been established for general use. Instead, several novel biomarkers have been proposed both to improve clinical prediction and to gain better insight into the pathogenesis of type 2 diabetes [12], [13]. These have produced promising results but the inferences have been limited by modest sample sizes, testing of one biomarker at a time and the lack of independent validation.

We have analyzed 31 novel biomarkers to test whether a single biomarker or a combined biomarker score could improve the prediction of clinically incident diabetes over and above the classical risk factors. We used a large, population-based cohort followed up for ten years to derive the prediction models and then validated the best predictors in another, independent cohort.

## Methods

### Cohort Descriptions

#### FINRISK97 Cohort

FINRISK97 involved 25–74 year old respondents to a survey conducted in five geographical areas in Finland [14]. It is based on a representative probability sample, drawn from the population register. The participants were instructed to fast for at least four hours before the scheduled examination and avoid heavy meals earlier that day. The median length of fasting was 5 hours (interquartile range 4–6 hours). The survey included a mailed questionnaire and a clinical examination. A blood sample was drawn for the measurement of serum lipids and gamma glutamyl transferase (GGT). Altogether, 8,444 persons participated and gave written informed consent. A detailed description of the cohort and methods is available on the MORGAM web-site at http://www.ktl.fi/publications/morgam/cohorts/full/finland/fin-fina.htm.

#### Health 2000 Cohort

Health 2000 was based on a stratified two-stage cluster sampling from the population register to represent the total Finnish population aged 30 years and over [15]. A detailed Methodology Report is available on the world-wide web (http://www.terveys2000.fi/doc/methodologyrep.pdf). The fasting instructions were similar to those of FINRISK97. The median length of fasting was 6.8 hours, interquartile range 5.6 – 13.5 hours). The survey included an interview on medical history, and health-related lifestyle habits, and a clinical examination. A blood sample was drawn from an antecubital vein. 6,200 persons participated and gave a written informed consent.

#### Ethics

Both FINRISK97 and Health 2000 studies were approved by the Ethics Committee of the National Public Health Institute and carried out according to the recommendations of the Declaration of Helsinki.

#### Diabetes at baseline

We used several data sources to ascertain cases of prevalent diabetes at baseline: (a) self-report of doctor-diagnosed diabetes or impaired glucose tolerance in the questionnaire, (b) the national drug reimbursement records and the National Hospital Discharge Register were checked for reimbursements of purchases of hypoglycemic drugs or hospitalizations with diabetes as the main or an additional diagnosis, and (c) blood glucose ≥7 mmol/L at baseline. If any of these sources was positive, the person was considered as having prevalent diabetes and was excluded from the analyses. Altogether 617 persons with prevalent diabetes were excluded in FINRISK97 and 7,827 persons were included in the analyses. In the Health 2000 Study, 1,224 persons were excluded either because of prevalent diabetes or age less than 35 or higher than 84 years. Thus, 4,976 persons were included in the analyses.

#### Follow-up for incident diabetes

The follow-up was until the end of 2007 for both cohorts. The median follow-up time was 10.8 years for the FINRISK97 and 7.1 years for the Health 2000 cohort. Clinically incident diabetes was the main outcome of interest. Three data sources were used to identify cases of clinically incident diabetes during the follow-up. (1) Record linkage of the study data with the National Drug Reimbursement Register on the basis of the personal identification code unique to each individual in the country. In Finland, persons with diabetes receive their hypoglycemic medications free of charge. To obtain this right, the person must present a statement from his/her physician documenting the clinical and laboratory findings that led to the diagnosis of diabetes. This statement is then reviewed by an expert physician of the National Social Insurance Institute and, if the documentation is found adequate, the right to the full reimbursement is granted. The Social Insurance Institute keeps a country-wide register of persons entitled to these reimbursements. (2) Record linkage with the National Hospital Discharge Register, which includes all hospitalizations in Finland (main diagnosis and up to four additional diagnoses). We checked whether diabetes (ICD-10 code E10-E14) was listed as any of the diagnoses for a hospitalization during the follow-up. (3) Record linkage with the National Causes-of-Death Register, which includes all deaths of permanent residents of Finland. We checked whether diabetes (ICD-10 code E10-E14) was mentioned as any of the causes of death (underlying cause of death, direct cause of death, or the contributing causes of death). If diabetes was found in any of these data sources, the person was considered to have incident diabetes. The date when the diabetes diagnosis first appeared was taken as the date of onset of diabetes. These procedures identify all cases of diabetes that were treated with hypoglycemic medications or hospitalized or who died during the follow-up. However, diabetic patients treated with diet only, who were not hospitalized and did not die, were not identified by these procedures.

In all, 417 cases of incident diabetes (249 in men and 168 in women) were observed in the FINRISK97 cohort and 179 cases (95 in men and 84 in women) in the Health 2000 cohort.

### Laboratory Methods

Most biomarkers were determined at the MORGAM Biomarker Laboratory, University of Mainz, Germany, from frozen, unthawed samples stored at −70°C, using methods that are described in more detail in the supporting information (Table S1). Apolipoprotein B100 (apoB), C-reactive protein (CRP), homocysteine and hemoglobin A1c (HbA1c) in the Health 2000 cohort as well as GGT in both cohorts were determined in the laboratories of the National Health and Welfare Institute, Turku and Helsinki, Finland, using routine commercial methods. Altogether, 31 biomarkers were determined in FINRISK97 and 10 were further determined in Health 2000 to replicate the findings on single biomarkers and to validate the performance of the biomarker score. Classic risk factors were determined locally using routine methods that have been described (http://www.terveys2000.fi/doc/methodologyrep.pdf) and [14].

### Statistical Methods

The analysis strategy comprised three stages: First, assessment of the associations between the single biomarkers and incident diabetes; second, assessment of the discriminative ability of single biomarkers in risk prediction models; and third, derivation of a composite biomarker score and its validation. In the first two stages, the assessment was done primarily in the FINRISK97 cohort and replicated in the Health 2000 cohort. In the third stage, the derivation of the biomarker score was based on the FINRISK97 cohort and it was validated in the Health 2000 cohort. We derived the biomarker score on the basis of: (a) the strength of association and reclassification in FINRISK; (b) considering biological plausibility; (c) correlations between the biomarkers to avoid including multiple biomarkers that reflect the same biological process, and (d) availability of the biomarkers in question in the validation cohort. Based on these criteria, we experimented with a few potential scores in the training sample taking into account both relative risk estimates and the net reclassification improvement by the score, and proceeded with the best ones to the validation sample.

The aim in model building was the prediction of absolute risk (10 years in FINRISK97 and 7 years in Health 2000). Discriminative ability of the models was tested using C-index [16] and the net reclassification improvement (NRI) and integrated discrimination improvement (IDI) statistics [17]. In reclassification analyses we used categories 0–4.9%, 5–9.9%, 10–19.9% and ≥20% in FINRISK97. Since the follow-up time was shorter for Health 2000, somewhat lower cutpoints were used: 0–2.9%, 3–7.9%, 8–14.9% and ≥15%. Model calibration was tested using the Hosmer-Lemeshow test with 10 risk groups. A more detailed description of statistical methods is presented in the supporting text (Text S1).

## Results

Both FINRISK97 and Health 2000 cohorts consisted of middle-aged persons with approximately equal numbers of men and women (Table 1). The levels of classic risk factors were as expected for a community-based middle-aged cohort. Geometric means of measured biomarkers are shown for both cohorts and both sexes in supporting information (Table S2). Correlation matrix between the biomarkers and classic risk factors is presented for FINRISK97 in supporting Table S3 and for Health 2000 in supporting Table S4.

**Table 1:** Baseline characteristics of study participants.

Characteristics	FINRISK97		HEALTH 2000	
	Men	Women	Men	Women
n (%)	3922 (50.1)	3905 (49.9)	2272 (45.7)	2704 (54.3)
Age (yrs)*	47.3 (37.8–60.9)	44.7 (35.6–57.0)	52.1 (43.9–61.7)	53.7 (44.3–64.5)
Body-mass index (kg/m^2^)*	26.6 (24.3–29.0)	25.8 (22.7–28.7)	26.8 (24.4–29.2)	26.3 (23.1–29.6)
Waist-Hip Ratio*	0.92 (0.88–0.97)	0.80 (0.75–0.84)	0.97 (0.94–1.01)	0.86 (0.82–0.90)
Systolic blood pressure (mmHg)*	137.9 (126.0–151.0)	130.3 (117.0–144.0)	134.7 (122.0–147.0)	132.6 (118.0–147.5)
Diastolic blood pressure (mmHg)*	83.7 (77.0–92.0)	79.4 (73.0–87.0)	84.1 (78.0–92.0)	79.9 (73.0–87.0)
Hypertension, n (%)	1389 (35.4)	1346 (34.5)	1159 (51.0)	1253 (46.3)
Current smoker, n (%)	1036 (26.4)	678 (17.4)	724 (31.9)	561 (20.7)
Prevalence of CVD, n (%)	305 (7.8)	111 (2.8)	188 (8.3)	154 (5.7)
High blood pressure medication, n (%)	501 (12.8)	390 (10.0)	463 (20.4)	685 (25.3)
Serum glucose (mmol/L)*	5.1 (4.7–5.4)	4.9 (4.6–5.2)	5.5 (5.2–5.8)	5.3 (5.0–5.6)
Total-cholesterol (mmol/L)*	5.4 (4.8–6.2)	5.4 (4.7–6.1)	6.0 (5.3–6.7)	5.9 (5.2–6.7)
LDL-cholesterol (mmol/L)*	3.4 (2.9–4.1)	3.2 (2.7–3.9)	3.9 (3.4–4.7)	3.8 (3.2–4.6)
HDL-cholesterol (mmol/L)*	1.2 (1.0–1.4)	1.5 (1.3–1.8)	1.2 (1.0–1.4)	1.4 (1.2–1.7)
Triglycerides (mmol/L)*	1.4 (1.0–2.0)	1.1 (0.8–1.5)	1.5 (1.1–2.1)	1.3 (0.9–1.6)

* Presented as geometric mean and interquartile range (Q1, Q3).

**Figure 1:**
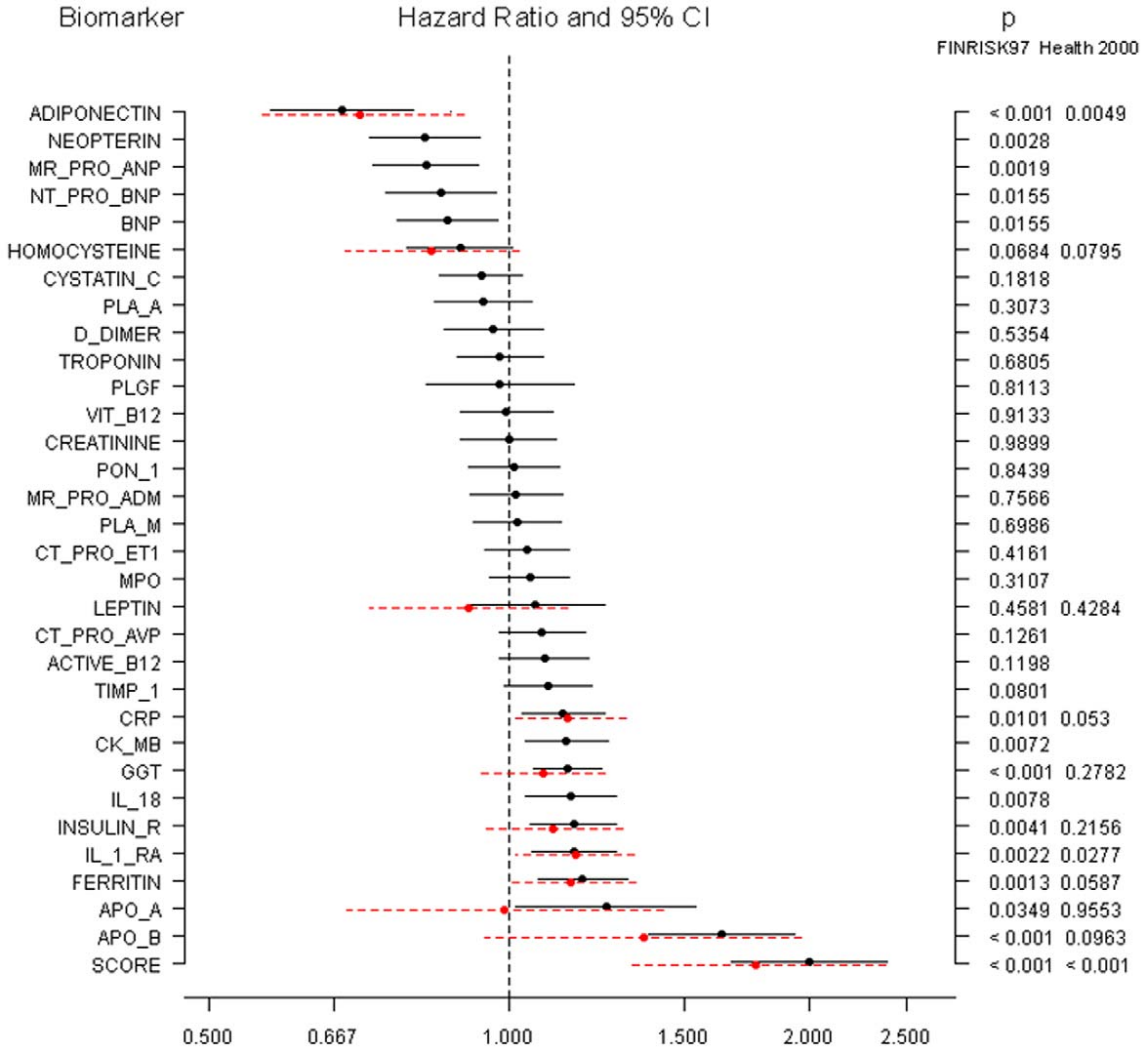
Hazard ratios (95% CI, per one SD) of clinically incident diabetes. FINRISK97 cohort (black solid lines) has 417 cases and 7,410 noncases. Selected biomarkers and the biomarker score were determined in the Health 2000 cohort (red dotted lines), which has 179 cases and 4,798 noncases. Data on men and women are combined. Adjusted for sex, non-HDL-cholesterol, HDL-cholesterol, triglycerides, BMI, systolic blood pressure, current smoking, blood glucose, history of a cardiovascular disease event and use of antihypertensive medication. Age was used as the time scale.

In Cox proportional hazards regression models controlling for classic risk factors (age as the time scale, sex, high density lipoprotein (HDL) cholesterol, non-HDL cholesterol, triglycerides, body mass index (BMI), systolic blood pressure, antihypertensive medication, current smoking, blood glucose, and history of CVD at baseline), four biomarkers were significantly associated with incident diabetes in both cohorts: adiponectin inversely, and CRP, interleukin-1 receptor antagonist (IL-1ra), and ferritin directly (Fig. 1). Furthermore, apoB was strongly associated with incident diabetes in FINRISK97 and had a borderline significant association (p = 0.053) in Health 2000. Additionally, seven other biomarkers were significantly associated with incident diabetes in FINRISK97. Two of them (GGT and insulin) were available for replication, but were nonsignificant in Health 2000 (Fig. 1). Sex-specific HRs are shown in supporting information (Figures S1 and S2).

To gain a better understanding of potential pathophysiological mechanisms, we further adjusted the model on ferritin for CRP. This did not reduce the HR substantially (from 1.18, p = 0.001, to 1.17, p = 0.002, in FINRISK97 and from 1.18, p = 0.031, to 1.17, p = 0.041, in Health 2000). Likewise, to control more fully for obesity, the model on apoB was further adjusted for waist-to-hip ratio. This had very little effect on HR (from 1.63, p = 9.2 e-9, to 1.58, p = 2.5 e-7, in FINRISK97 and from 1.52, p = 0.053, to 1.50, p = 0.057, in Health 2000). We also carried out a sensitivity analysis adjusting for physical activity, but HRs of the novel biomarkers remained essentially unchanged, although physical activity itself was clearly protective.

For comparison it should be noted that the HR for BMI, after adjusting for other classic risk factors, was in FINRISK97 1.95 (p = 7.4 e-66). Interestingly, even after adjusting for blood glucose and other classic risk factors, HbA1c was strongly associated with incident diabetes in Health 2000 (HR = 2.35, 95%CI 1.97 – 2.79, p = 6.2 e-19).

### Discrimination

Adiponectin, interleukin-18 (IL-18) and insulin improved the C-index significantly, albeit modestly, in FINRISK97. None of the single biomarkers improved C-index in Health 2000. Adiponectin, apoB, CK-MB, CRP, ferritin and IL-18 improved IDI in FINRISK97, but none of them replicated in Health 2000.

### Reclassification

The addition of single biomarkers to the classic risk factors in FINRISK97, revealed that nine biomarkers improved classification significantly. The strongest were apoB (NRI = 8.7%, p<0.0001) and adiponectin (NRI = 6.7%, p = 0.005). However, no single biomarker improved classification in Health 2000.

### Biomarker score and its validation in Health 2000 cohort

**Figure 2:**
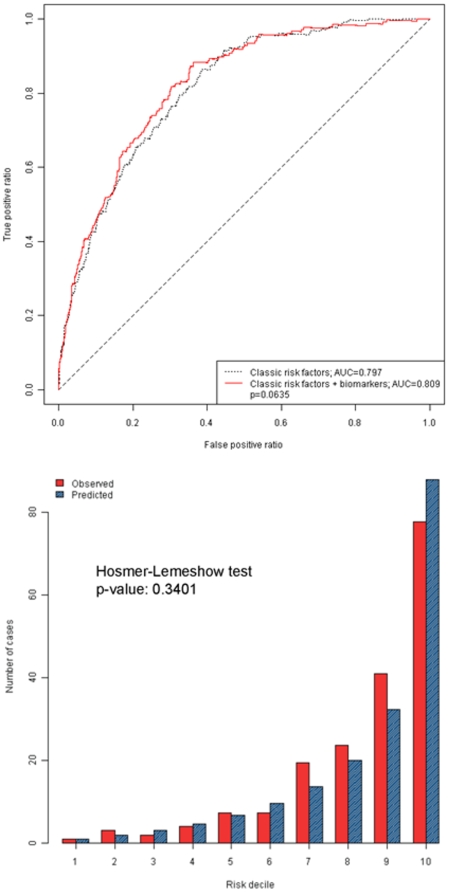
ROC curves and C-index with and without the four biomarker score, and calibration of the model with the four biomarker score. Health 2000 study. The score includes adiponectin, apoB, CRP, and ferritin. The model is adjusted for the same classic risk factors as in Fig. 1. Age was used as the time scale.

Based on the FINRISK97 results, we created a biomarker score which, for men and women combined, consisted of a linear combination of adiponectin, apoB, CRP, and ferritin. In Health 2000 this score was associated with incident diabetes with an adjusted HR of 1.88 (1.40 – 2.53, p = 2.8 e-5) (Fig. 1). The score also improved IDI significantly (change in IDI = 0.0149, p<0.0001), but the improvement in C-index did not quite reach statistical significance (p = 0.064) (Fig. 2a). The NRI was, however, significant 11.8% (p = 0.0061) (Table 2) [18], [19]. Calibration of the prediction model was good (Fig. 2b). An analysis by gender suggested that the best score differed between men and women. Among men, the best results were obtained with the score of four biomarkers: adiponectin, apoB, ferritin and IL-1ra, which gave an NRI of 25.4% (p<0.0001) (supporting information, Table S5). The corresponding IDI was 0.0432 (p<0.0001) and the C-index also improved significantly from 0.784 to 0.828 (p = 0.002). Among women, the best results were obtained with the score including four biomarkers, adiponectin, apoB, CRP and insulin. This score gave an NRI of 13.6% (p = 0.041) (Supporting information, Table S5). IDI was also significant, 0.0188 (p = 0.003) but the change in C-index remained modest and nonsignificant (p = 0.277) among women. Equations for the scores in men, women and both genders combined are presented in supporting information (Table S6).

**Table 2:** Net reclassification improvement* due to the biomarker score†.

	Predicted risk with biomarker score					
Persons developing diabetes during 7-year follow-up (n = 174)	<3%	3–7.9%	8–14.9%	≥15%	up‡	down‡
<3%	29 (75.2%)	8 (22.2%)	1 (2.6%)	0 (0.0%)		
3–7.9%	8 (11.0%)	48 (67.5%)	14 (19.4%)	1 (2.1%)	39 (21.0%)	22 (12.0%)
8–14.9%	0 (0.0%)	10 (23.6%)	17 (41.6%)	14 (34.8%)		
≥15%	0 (0.0%)	1 (3.3%)	4 (9.4%)	33 (87.2%)		
Persons not developing diabetes during 7-year follow-up (n = 4803)						
<3%	3000 (95.8)	132 (4.2%)	0 (0.0%)	0 (0.0%)		
3–7.9%	326 (27.0%)	766 (63.5%)	113 (9.4%)	2 (0.1%)	297 (6.2%)	433 (9.0%)
8–14.9%	3 (1.0%)	80 (25.6%)	180 (57.3%)	51 (16.2%)		
≥15%	1 (0.7%)	4 (2.7%)	18 (13.5%)	113 (83.0%)		

* Net reclassification improvement 11.8% (SE 0.043), p = 0.0061.

† The biomarker score consists of adiponectin, apolipoprotein B, C-reactive protein and ferritin. The conventional risk factor model included the same risk factors as in Fig. 1. Coefficients from FINRISK97 are applied to the Health 2000 validation cohort. Men and women combined.

‡ The numbers of persons reclassified up and down do not exactly equal to the sum of different categories, because the reclassification analysis has been performed using the Kaplan-Meier approach and the result has been rounded to the nearest integer.

## Discussion

Without any doubt, obesity is the strongest single predictor of diabetes risk in middle aged individuals. Our study showed, however, that adiponectin, apoB, CRP, and ferritin improved the prediction of diabetes consistently in two independent cohorts even after taking BMI, blood glucose and other classic risk factors into account. Data suggested even more substantial improvements in gender-specific analyses. Among men, the score consisting of four biomarkers, adiponectin, apoB, IL-1ra and ferritin, improved net reclassification by 25% and measures of model discrimination also improved clearly. Among women, the best score consisted of adiponectin, apoB, CRP anf insulin, and gave an NRI of 14%. Suggestive evidence was found in the FINRISK97 cohort for seven other biomarkers, which may deserve further research. These findings may help to identify persons at high risk of diabetes and improve the targeting of preventive measures. Perhaps more importantly, they suggest pathophysiological pathways leading to diabetes in middle-aged individuals and these pathways may also be amenable to intervention.

Earlier attempts at creating algorithms for the prediction of diabetes have mainly focused on routinely measured clinical risk factors [8], [9], [20], [21]. Recently, however, Kolberg and coworkers reported a case control study nested in a life-style intervention trial on cardiovascular diseases[12]. They tested a panel of 58 biomarkers in 160 cases and 472 controls and found that six biomarkers (adiponectin, CRP, ferritin, interleukin-2 receptor A, glucose and insulin) helped to predict the 5-year risk of incident diabetes. Many of these biomarkers are the same as in our study, even though we added a validation in an independent cohort to avoid overoptimism. Two other recent papers evaluated a set of genetic variants in addition to the clinical risk factors [10], [11]. In the Framingham Offspring Study NRI remained modest, 4.1% to 2.1%, depending on the model, but in the Malmö Preventive Project an NRI of 9% and in the Botnia project an NRI of 20% (p = 0.05) was achieved.

Several studies have tested single biomarkers in addition to the classic risk factors, usually using a nested case-control design. By far the most data exist on CRP, which has been associated with future diabetes in multiple studies[12], [22], [23]. In agreement with the present study, high adiponectin has predicted a low risk of diabetes in different populations [24], [25]. Serum ferritin concentration has been found to be an indicator of diabetes risk in the European Prospective Investigation of Cancer (EPIC)-Norfolk Study [26], the Nurses Health Study [27] and the Atherosclerosis Risk in Communities (ARIC) Study [28]. In the latter study, however, adjustment for BMI abolished the association. To distinguish between the acute phase response and the iron metabolism, we further adjusted for CRP, which did not reduce the HR of ferritin substantially, supporting the concept that the ferritin-diabetes association may not reflect the acute phase response but mainly the iron metabolism. Interleukin-18 was significantly associated with increased risk of diabetes in the German MONICA-KORA study, which is in agreement with our findings in FINRISK97 [29]. Unfortunately, we did not have interleukin-18 available for replication in Health 2000.

IL-1ra is an interesting cytokine, which was associated with incident type 2 diabetes in a recent case-control analysis of the Whitehall II Study [30]. In a clinical trial, recombinant IL-1ra improved beta-cell function and glycemic control in patients with type 2 diabetes [31]. In our study, Il-1ra was consistently associated with incident diabetes in both cohorts, which agrees with these earlier reports. Somewhat surprisingly, one of the strongest predictors of diabetes was apoB. The most obvious explanation for this association would be obesity, but controlling for both BMI and waist- to-hip ratio did not reduce the association substantially. The ‘common soil’ hypothesis suggests that diabetes and CVD share common antecedents [32]. The possibility that apoB could play a role in both seems to deserve more detailed study.

The biomarkers identified in our study suggest at least three interesting areas in the pathophysiology of diabetes, which warrant further research. First, adiponectin is emerging as a potent antidiabetic hormone. It is produced and secreted by adipocytes but is inversely correlated with obesity. It increases insulin sensitivity, improves glucose tolerance and inhibits inflammation. However, the associations of adiponectin with cardiovascular and total mortality are controversial, the majority of studies seem to support increased, rather than decreased risk [12], [33]. Secondly, ferritin was associated with increased risk of diabetes and the association was particularly evident among men, whereas no significant association was observed among women. This, together with the fact that the association was robust to adjustment for CRP, suggests a role for iron overload in the pathogenesis of diabetes. Third, IL-1Ra was associated with increased risk of diabetes. IL-1Ra is an anti-inflammatory cytokine, but its elevation may be compensatory to the increased production of proinflammatory IL-1 beta in the pancreas, which is known to induce beta cell apoptosis and impair insulin secretion [28], [34].

The strengths of our study include a simultaneous evaluation of a large panel of biomarkers, large cohorts, a prospective population-based design and the validation of prediction in an independent cohort. Certain limitations should also be mentioned. First, even though we had altogether 590 cases of clinically incident diabetes in our study, we had only 179 incident cases in the validation cohort available for the analyses on seven-year absolute risk of diabetes. Therefore, the numbers in gender-specific analyses were smallish and these results need to be confirmed in future studies. Secondly, the age range in the validation cohort was wide, 35–84 years, which may have attenuated the results since age alone is a strong risk factor and the risk prediction usually works best in middle-aged individuals. Thirdly, we could not analyze all 31 biomarkers in the Health 2000 cohort. We chose the most promising ones for replication but, nevertheless, there were five biomarkers with significant HRs in FINRISK97 that were not available for replication in Health 2000. Fourthly, we did not carry out oral glucose tolerance tests at baseline or measure fasting blood glucose at a follow-up examination. Our outcome was clinically incident diabetes, identified through the use of hypoglycemic medications, diagnoses for hospitalizations and causes of death. Therefore, we could not identify clinically mild cases of diabetes, treated with diet only. This may have reduced the statistical power slightly, but it is unlikely that the predictors would have been different if we could have included cases of diabetes treated with diet only.

In conclusion, after accounting for classic risk factors, our study identified adiponectin, apoB, CRP, IL-1ra and ferritin as the strongest predictors of incident diabetes. The biomarker score, composed as a linear combination of four biomarkers, was associated with doubling of the relative hazard of diabetes in the independent validation cohort. The prediction of absolute risk of diabetes produced a significantly improved net reclassification and discrimination, especially in gender-specific analyses, with the model including the biomarker score. This information may help with identifying individuals at high risk of developing diabetes. Perhaps more importantly, it may indicate directions which further research on the pathogenesis and prevention of diabetes should take.

## Supporting Information

Text S1 (0.33 MB DOC)

Table S1 Methods for biomarker determinations and quality control results.
(0.11 MB DOC)

Table S2 Geometric means (inter-quartile range) of biomarkers analyzed in the FINRISK97 and Health 2000 cohorts.
(0.10 MB DOC)

Table S3 Rank correlation matrix. FINRISK97, men and women combined.
(0.43 MB DOC)

Table S4 Rank correlation matrix. Health 2000, men and women combined.
(0.14 MB DOC)

Table S5 Gender-specific net reclassification improvement (NRI) due to the biomarker score. Coefficients from FINRISK97 are applied to the Health 2000 validation cohort.
(0.06 MB DOC)

Table S6 Equations for the best biomarker scores for men, women, and men and women combined
(0.02 MB DOC)

Figure S1 Hazard ratios (95% CI, per one SD) of clinically incident diabetes among men. FINRISK97 cohort (black solid lines) has 249 cases and 3,673 noncases among men. Selected biomarkers and the biomarker score were determined in the Health 2000 cohort (red dotted lines), which has 95 cases and 2,178 noncases. Adjusted for non-HDL-cholesterol, HDL-cholesterol, triglycerides, BMI, systolic blood pressure, current smoking, blood glucose, history of a cardiovascular disease event and use of antihypertensive medication. Age was used as the time scale. The biomarker score for men included adiponectin, apoB, ferritin and IL-1ra.
(3.74 MB TIF)

Figure S2 Hazard ratios (95% CI, per one SD) of clinically incident diabetes among women. FINRISK97 cohort (black solid lines) has 168 cases and 3,737 noncases among women. Selected biomarkers and the biomarker score were determined in the Health 2000 cohort (red dotted lines), which has 84 cases and 2,620 noncases. Adjusted for non-HDL-cholesterol, HDL-cholesterol, triglycerides, BMI, systolic blood pressure, current smoking, blood glucose, history of a cardiovascular disease event and use of antihypertensive medication. Age was used as the time scale. The biomarker score for women included adiponectin, apoB, CRP, and insulin.
(0.35 MB TIF)
