# Supplementary material for: Thirty-One Novel Biomarkers as Predictors for Clinically Incident Diabetes
Source: PLoS One. 2010 Apr 9;5(4):e10100. doi: 10.1371/journal.pone.0010100 (PMC2852424; doi:10.1371/journal.pone.0010100)
Supplement: Text S1 — (0.33 MB DOC) [file pone.0010100.s001.doc]

**Supporting Text S1**

Supplement to: Veikko Salomaa, Aki S. Havulinna, Olli Saarela, Tanja Zeller, Pekka Jousilahti, Antti Jula, Thomas Münzel, Arpo Aromaa, Alun Evans, Kari Kuulasmaa, Stefan Blankenberg. **Thirty-One Novel Biomarkers as Predictors for Clinically Incident Diabetes.**

**Statistical methods**

In FINRISK97 the proportion of missing data due to sample unavailability or other reasons varied from 0.7% to 13% for different biomarkers, but was larger for troponin (24.3%) and for blood glucose (36.0%). In Health 2000, the proportion of missing data was small varying from 0% to 1.1% for different biomarkers. Since several of the 31 biomarkers had some amount of missing data, using only participants with complete data would have led to notable loss of information in multivariate analyses. Also, discarding the incomplete observations may cause bias if the sample availability is related to the disease endpoints of interest. For these reasons we opted to use multiple imputation [1] [2] to handle the missing data in all of our analyses. The imputation model included all biomarkers, classic risk factors and disease status at the start of the follow-up and at the end of the follow-up. Using the disease status in the imputation is important to avoid attenuation of the estimated effects [3]. This was especially important due to varying proportion of missing values in different markers (e.g. due to different availability of plasma/serum samples), as omitting the outcome would have resulted in more severe attenuation for markers with more missing values. The imputation model was a multinormal regression model where the biomarker variables were normalised using the formula
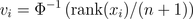
, where Ф is the cumulative distribution function of the standard normal distribution. Imputation was carried out using WinBUGS software [4]. The imputed values from the multinormal model were transformed back to the original scale using inverses of the empirical cumulative distribution functions. Using this procedure we avoided the need to make parametric assumptions about the biomarker distributions at the imputation stage. Due to technical reasons, some biomarker measurements were below or above the detection limits. In principle it would be possible to use multiple imputation also for the undetectable values. However, since the proportion of undetectable values was usually small, and because we wanted to avoid making parametric assumptions about the tails of the distributions, we opted to use the detection limits in place of the undetectable values. We corrected the ‘semi-fasting’ blood glucose and insulin values using a linear effect for time of day and penalized spline smoothing for the effect of the length of fasting, plus an offset term for the fasting <1 hour. The corrected values were used in the analyses.

Associations of single biomarkers and disease endpoints were investigated with Cox regression models adjusted for classic risk factors (HDL cholesterol, non-HDL cholesterol, BMI, systolic blood pressure, daily smoking, and use of cholesterol and blood pressure medication). In all time-to-event models age was used as the time scale. Models for incident diabetes excluded individuals with prevalent diabetes or self-reported impaired glucose tolerance at baseline. In a population sample the distributions of many of the markers are very skewed to the right. To study the functional form of the associations, we experimented with different transformations and spline fits for the biomarker variables. In many cases root transformations resulted in a better fit in the proportional hazards model than normalising or logarithmic transformations. This seems natural since the distributions are limited to zero, and the latter transformations needlessly spread out the left tail of the distributions. We ended up using cubic root transformations for distributions with skewness > 2 while using the other markers on their original scale. Time-to-event models were fitted using the "survival" package of R statistical software [5].

The aim in model building was to compare absolute risks (10-year risk in FINRISK97 and 7-year risk in Health 2000) predicted by models with biomarkers included to a baseline model which included only classic risk factors. When comparing models derived from the same data, effects of overfitting were avoided by calculating the predicted risks using 10-fold cross-validation. In this procedure the data was randomly divided into ten groups and predictions for each tenth were calculated from a model estimated using the other nine tenths. Because baseline hazard estimates derived from a Cox model may be unreliable at the boundaries of the data, predicted absolute risks were calculated from the Weibull curve fitted over age, adjusting for the linear predictor estimated from the respective Cox model. Discriminative ability of the models was tested using c-index improvement [6] and the NRI and IDI test statistics [7]. Model calibration was tested using the Hosmer-Lemeshov test with 10 risk groups. Reclassification and calibration statistics, as well as ROC-curves, were calculated following the Kaplan-Meier approach [8] [9], where censoring in time-to-event data is taken into account.

**References**

1. Rubin DB. Multiple imputation for nonresponse in surveys. J. Wiley & Sons, New York, 1987.
2. He Y. Missing data analysis using multiple imputation. Getting to the heart of the matter. Circ Cardiovasc Qual Outcomes 2010;3:98-105.
3. Moons KG, Donders RA, Stijnen T, Harrell Jr FE. Using the outcome for imputation of missing predictor values was preferred. J Clinl Epidemiol 2006; **59**: 1092–1101.
4. Lunn DJ, Thomas A, Best N, Spiegelhalter D. WinBUGS – a Bayesian modelling framework: concepts, structure, and extensibility. *Stat Comput* 2000; **10**: 325–337.
5. R Development Core Team. R: A language and environment for statistical computing. Vienna, Austria: R Foundation for Statistical Computing. [http://www.R-project.org](http://www.R-project.org/), 2006.
6. Antolini L, Nam B-H, D'Agostino RB. Inference on correlated discrimination measures in survival analysis: a nonparametric approach. *Commun Stat Theory Methods* 2004; **33**: 2117–35.
7. Pencina MJ, D’Agostino Sr RB, D’Agostino Jr RB, Vasan RS. Evaluating the added predictive ability of a new marker: From area under the ROC curve to reclassification and beyond. Stat Med 2008; **27**: 157–72.
8. Heagerty PJ, Lumley T, Pepe MS. Time-dependent ROC curves for censored survival data and a diagnostic marker. Biometrics 2000; **56**: 337–44.
9. Steyerberg EW, Pencina MJ. Reclassification calculations for persons with incomplete follow-up. (Letter). Ann Intern Med 2010;152:195-6.

| Supporting Table S1: Methods for biomarker determinations and quality control results. |  |  |  |
| --- | --- | --- | --- |
|  |  |  |  |
|  |  |  |  |
|  |  |  |  |
|  |  |  |  |
|  |  |  |  |
|  |  |  |  |
|  |  |  |  |
|  |  |  |  |
|  |  |  |  |
|  |  |  |  |
|  |  |  |  |
|  |  |  |  |
|  |  |  |  |
|  |  |  |  |
|  |  |  |  |
|  |  |  |  |
|  |  |  |  |
|  |  |  |  |
|  |  |  |  |
|  |  |  |  |
|  |  |  |  |
|  |  |  |  |
|  |  |  |  |
|  |  |  |  |
|  |  |  |  |
|  |  |  |  |
|  |  |  |  |
|  |  |  |  |
|  |  |  |  |
|  |  |  |  |

|  |  | | |
| --- | --- | --- | --- |
|  |  |  |  |
|  |  |  |  |
|  |  |  |  |
|  |  |  |  |
|  |  |  |  |
|  |  |  |  |
|  |  |  |  |
|  |  |  |  |
|  |  |  |  |
|  |  |  |  |
|  |  |  |  |
|  |  |  |  |
|  |  |  |  |
|  |  |  |  |
|  |  |  |  |
|  |  |  |  |
|  |  |  |  |
|  |  |  |  |
|  |  |  |  |

|  |  | | |
| --- | --- | --- | --- |
|  |  |  |  |
|  |  |  |  |
|  |  |  |  |
|  |  |  |  |
|  |  |  |  |
|  |  |  |  |
|  |  |  |  |
|  |  |  |  |
|  |  |  |  |
|  |  |  |  |
|  |  |  |  |
|  |  |  |  |
|  |  |  |  |

ohorts.

|  |  |  |  |
| --- | --- | --- | --- |
|  |  |  |  |
|  |  |  |  |
|  |  |  |  |
|  |  |  |  |
|  |  |  |  |
|  |  |  |  |
|  |  |  |  |
|  |  |  |  |
|  |  |  |  |
|  |  |  |  |
|  |  |  |  |
|  |  |  |  |
|  |  |  |  |
|  |  |  |  |
|  |  |  |  |
|  |  |  |  |
|  |  |  |  |
|  |  |  |  |
|  | |  |  |
|  |  |  |  |
|  |  |  |  |
|  |  |  |  |
|  |  |  |  |
|  |  |  |  |
|  |  |  |  |
|  |  |  |  |
|  |  |  |  |
|  |  |  |  |
|  |  |  |  |
|  |  |  |  |
|  |  |  |  |
|  |  |  |  |
|  |  |  |  |
|  |  |  |  |
|  | |  |  |
|  |  |  |  |
|  |  |  |  |
|  |  |  |  |
|  |  |  |  |
|  |  |  |  |
|  |  |  |  |
|  |  |  |  |
|  |  |  |  |
|  |  |  |  |
|  |  |  |  |
|  |  |  |  |
|  |  |  |  |
|  |  |  |  |
|  |  |  |  |
|  |  |  |  |
|  |  |  |  |
|  |  |  |  |
|  |  |  |  |
|  |  |  |  |
|  |  |  |  |
|  |  |  |  |
|  |  |  |  |
|  |  |  |  |
|  |  |  |  |
|  | |  |  |
|  |  |  |  |
|  |  |  |  |
|  |  |  |  |
|  |  |  |  |
|  |  |  |  |

|  |  | | | | | |
| --- | --- | --- | --- | --- | --- | --- |
|  |  |  |  |  |  |  |
|  |  |  |  |  |  |  |
|  |  |  |  |  |  |  |
|  |  |  |  |  |  |  |
|  |  |  |  |  |  |  |
|  |  |  |  |  |  |  |
|  |  |  |  |  |  |  |
|  |  |  |  |  |  |  |
|  |  |  |  |  |  |  |
|  |  |  |  |  |  |  |
|  |  |  |  |  |  |  |
|  |  |  |  |  |  |  |
|  |  |  |  |  |  |  |
|  |  |  |  |  |  |  |
|  |  |  |  |  |  |  |
|  |  |  |  |  |  |  |
|  |  |  |  |  |  |  |
|  |  |  |  |  |  |  |
|  |  |  |  |  |  |  |
|  |  |  |  |  |  |  |
|  |  |  |  |  |  |  |
|  |  |  |  |  |  |  |
|  |  |  |  |  |  |  |

|  |
| --- |
|  |
|  |

|  |  |  |  |  |
| --- | --- | --- | --- | --- |
|  |  |  |  | |

|  |  |  |  |  |
| --- | --- | --- | --- | --- |
|  |  |  |  | |
