# Supplementary material for: Thirty-One Novel Biomarkers as Predictors for Clinically Incident Diabetes
Source: PLoS One. 2010 Apr 9;5(4):e10100. doi: 10.1371/journal.pone.0010100 (PMC2852424; doi:10.1371/journal.pone.0010100)
Supplement: Table S1 — Methods for biomarker determinations and quality control results. (0.11 MB DOC) [file pone.0010100.s002.doc]

| **Biomarker (Unit)** |  | **Method (Company, Instrument)** | **Material** | **Procedure** | Intra-assay^+^ CV (%) | Inter-assay^+^  CV (%) |
| --- | --- | --- | --- | --- | --- | --- |
| Adiponectin (ng/mL) |  | ELISA (R&D) | Plasma (EDTA) | R&D Assay-internal standards 1-8 (0-25,000 ng/mL)* within each 96well-plate  Laboratory-internal Plasma-Control within each 96well-plate | 4.49 | 16.68 |
| Apolipoprotein A1 (g/L) | Apo A | Immunoturbidimetric assay (Abbott; Architect *c*8000) | Serum | BioRad Liquichek Immunology Controls high/medium/low (high:2.31 g/L; medium:1.57 g/L; low:0.835 g/L)* every 24h  Laboratory-internal Serum-Control sample every 24h | 2.19 | 1.44 |
| Apolipoprotein B (g/L) | Apo B | Immunoturbidimetric assay (Abbott, Architect *c*8000) | Serum | BioRad Liquichek Immunology Controls high/medium/low (high:1.45 g/L; medium:0.951 g/L; low:0.451 g/L)* every 24h  Laboratory-internal Serum-Control sample every 24h | 2.0 | 2.22 |
| B-type natriuretic peptide (pg/mL) | BNP | Chemiluminescent microparticle immunoassay CMIA (Abbott, Architecht *i*2000) | Plasma (EDTA) | Abbott BNP Controls low/medium/high (high:3,500 pg/mL; medium:500 pg/mL; low:90 pg/mL)*  Laboratory-internal Plasma-Control sample every 24h | 2.11 | 4.28 |
| Copeptin (pmol/L) | CT-proAVP | Immunoluminometric assay CT-proAVP (B.R.A.H.M.S, ELISA) | Plasma (EDTA) | BRAHMS Assay-internal standards 1-5 (0-478 pmol/L)* within each 96well-plate  BRAHMS Controls 1+2 (1.2 pmol/L and 12 pmol/L) within each 96well-plate  Laboratory-internal Plasma-Control within each 96well-plate | 3.65 | 2.33 |
| C-reactive protein (mg/L) | CRP | Latex immunoassay CRP16 (Abbott, Architect *c*8000) | Serum | Sentinel Diagnostics Immuno Controls high/medium/low (high:57.9 mg/L; medium:16.6 mg/L; low:0.5 mg/L)* every 24h  Laboratory-internal Serum-Control sample every 24h | 0.93 | 0.83 |
| C-terminal pro-endothelin-1 (pmol/L) | CT-pro ET-1 | Immunoluminometric assay CT-proET1 (B.R.A.H.M.S, Kryptor) | Plasma (EDTA) | BRAHMS CT-pro ET1 QC 1+2 (1: 65.3 pmol/L, 2: 187 pmol/L) every 24h  Laboratory-internal Plasma-Control sample every 24h | 2.61 | 3.57 |
| Cystatin C (mg/L) |  | Latex immunoassay (Abbott, Architect *c*8000) | Plasma (EDTA) | Sentinel Diagnostics Cystatin C Controls low/high (high: 0.85 mg/mL, low: 4.08 mg/L)* every 24h  Laboratory-internal Plasma-Control sample every 24h | 3.34 | 1.8 |
| Creatinine (mg/dL) |  | Kinetic Assay (Abbott, Architect *c*8000) | Serum | BioRad Lyphochek Assayed Chemistry Controls high/ /low (high:4.54 mg/dL; low:1.53 mg/dL)* every 24h  Laboratory-internal Serum-Control sample every 24h | 0.09 | 2.28 |
| Creatine-Kinase MB (ng/mL) | CK-MB | Chemiluminescent microparticle immunoassay CMIA (Abbott. Architect *i*2000) | Serum | Abbott STAT CK-MB Controls low/medium/high every 24 h  Laboratory-internal Serum-Control sample every 24h | 4.9 | 11.43 |
| D-Dimer (ng/mL) |  | Microparticle enzyme immunoassay MEIA (Abbott, Architect *c*8000) | Plasma (EDTA) | Abbott Quantia D-Dimer Control 1+2 (1:458 ng/mL; 2:891 ng/mL)* every 24h  Laboratory-internal Plasma-Control sample every 24h | 0.95 | 2.05 |
| Ferritin (ng/L) |  | Chemiluminescent microparticle immunoassay CMIA (Abbott, Architect *i*2000) | Serum | Abbott Ferritin Controls low/medium/high (high:400 ng/L; medium: 150 ngl/L, low:20 ng/L)* every 24h  Laboratory-internal Serum-Control sample every 24h | 3.19 | 3.9 |
| Homocysteine (µmol/L) |  | Fluorescence polarization immunoassay FPIA (Abbott, AxSYM) | Serum | Abbott AxSYM Homocysteine Controls high/medium/low (high:25 µmol/L; medium: 12.5 µmol/L, low:7 µmol/L)* every 24h  Laboratory-internal Serum-Control sample every 24h | 3.43 | 5.04 |
| Interleukin-1 Receptor Antagonist (pg/mL) | IL-1Ra | ELISA (R&D) | Serum | R&D Assay-internal standards 1-8 (0-2,000)* within each 96well-plate  Laboratory-internal Serum-Control sample every 24h | 3.59 | 5.68 |
| Interleukin-18 (pg/mL) | IL-18 | ELISA (MBL) | Serum | MBL Assay-internal standards 1-6 (0-5,000 pg/mL)* within each 96well-plate  Laboratory-internal Serum-Control within each 96well-plate | 6.98 | 12.62 |
| Insulin (µU/mL) |  | Chemiluminescent microparticle immunoassay CMIA (Abbott, Architect *i*2000) | Serum | Abbott Insulin Controls low/medium/high (high:120 µU/mL; medium: 40 µU/mL, low:8 µU/mL)* every 24h  Laboratory-internal Serum-Control sample every 24h | 3.05 | 3.31 |
| Leptin (pg/mL) |  | ELISA (R&D) | Serum | R&D Assay-internal standards 1-8 (0-100,000 pg/mL)* within each 96well-plate  Laboratory-internal Serum-Control sample every 24h | 5.41 | 8.93 |
| Midregional-pro adrenomedullin (nmol/L) | MR-proADM | Immunoluminometric assay MR-proADM (B.R.A.H.M.S, Kryptor) | Plasma (EDTA) | BRAHMS MR-proADM QC 1+2 (1: 0.73 nmol/L, 2: 3.79 nmol/L) every 24h  Laboratory-internal Plasma-Control sample every 24h | 2.17 | 2.43 |
| Midregional-pro atrial natriuretic peptide (pmol/L) | MR-pro ANP | Immunoluminometric assay MR-proANP (B.R.A.H.M.S, Kryptor) | Plasma (EDTA) | BRAHMS MR-proANP QC 1+2 (1:92.3 pmol/L, 2:474 pmol/L)* every 24h  Laboratory-internal Plasma-Control sample every 24h | 3.65 | 2.33 |
| Myeloperoxidase (µg/L) | MPO | Chemiluminescent microparticle immunoassay CMIA (Abbott, Architect *i*2000) | Plasma (EDTA) | Abbott MPO Controls low/medium/high (high:551 µg/L; medium:174 µg/L; low:58 µg/L)* every 24h  Laboratory-internal Plasma-Control sample every 24h | 6.15 | 4.09 |
| Neopterin (nmol/L) |  | ELISA Neopterin (B.R.A.H.M.S, ELISA) | Plasma (EDTA) | BRAHMS Assay-internal standards 1-6 (2-250 nmol/L)* within each 96well-plate  BRAHMS Controls 1+2 (1:12pmol/L, 2:103 pmol/L)* within each 96well-plate  Laboratory-internal Plasma-Control within each 96well-plate | 3.03 | 6.57 |
| N-terminal-pro B-type natriuretic peptide (pg/mL) | NT-proBNP | Electrochemiluminescence sandwich immunoassay ECLIA  (Roche Diagnostics, Elecsys 2010) | Serum | Roche Elecsys PreciControl CardiacII PC CARD 1+2 (1: ~150 pg/mL; 2: ~5000 pg/mL)*  Laboratory-internal Serum-Control sample every 24h | 2.58 | 1.38 |
| Lipoprotein-associated phospholipase A_2_ (Mass) (ng/mL) | Lp-PLA_2_ | ELISA Lp-PLA_2_ PLAC (diaDexus) | Plasma (EDTA) | DiaDexus Assay-internal standards 1-6 (0-1,000 ng/mL)* within each 96well-plate  DiaDexus Lp-PLA_2_ PLAC Controls 1+2 (~150 ng/mL and ~400 ng/mL) within each 96well-plate  Laboratory-internal Plasma-Control within each 96well-plate | 4.58 | 9.14 |
| Lipoprotein-associated phospholipase A_2_ (Activity) (nmol/min/mL) | Lp-PLA_2_ | Colorimetric activity assay CAM (diaDexus) | Plasma (EDTA) | DiaDexus p-Nitrophenol Calibration Curve (0-100 nmoles) every 72h  DiaDexus Lp-PLA_2_ CAM Controls high/low (high:635-924 nmole/min/mL; low:174-221 nmole/min/mL)* within each 96well-plate  Laboratory-internal Plasma-Control sample within each 96well-plate | 2.1 | 7.9 |
| Placental growth factor (cardiac) (pg/mL) | cPlGF | Chemiluminescent microparticle immunoassay CMIA (Abbott, Architect *i*2000) | Plasma (EDTA) | Abbott *c*PlGF Controls high/medium/low (high:175 pg/mL; medium:40 pg/mL; low:15 pg/mL)* every 24h  Laboratory-internal Plasma-Control sample every 24h | 3.79 | 3.0 |
| Paraoxonase-1 activity (nmol/min/mL) | PON-1 | Activity assay  [Charlton-Menys V](http://www.ncbi.nlm.nih.gov/sites/entrez?Db=pubmed&Cmd=Search&Term="Charlton-Menys V"%5BAuthor%5D&itool=EntrezSystem2.PEntrez.Pubmed.Pubmed_ResultsPanel.Pubmed_DiscoveryPanel.Pubmed_RVAbstractPlus), [Liu Y](http://www.ncbi.nlm.nih.gov/sites/entrez?Db=pubmed&Cmd=Search&Term="Liu Y"%5BAuthor%5D&itool=EntrezSystem2.PEntrez.Pubmed.Pubmed_ResultsPanel.Pubmed_DiscoveryPanel.Pubmed_RVAbstractPlus), [Durrington PN](http://www.ncbi.nlm.nih.gov/sites/entrez?Db=pubmed&Cmd=Search&Term="Durrington PN"%5BAuthor%5D&itool=EntrezSystem2.PEntrez.Pubmed.Pubmed_ResultsPanel.Pubmed_DiscoveryPanel.Pubmed_RVAbstractPlus). Semiautomated method for determination of serum paraoxonase activity using paraoxon as substrate.  *Clin Chem* 2006; **52**: 453-7. | Serum | Control samples high/low (high:177.5-257.9 nmole/ml/min; low:28-53 nmole/ml/min)^†^ within each 96well-plate  Laboratory-internal Serum-Control samples within each 96well-plate | 3.29 | 4.53 |
| Tissue inhibitor of metalloproteinase 1 (ng/mL) | TIMP-1 | Chemiluminescent microparticle immunoassay CMIA (Abbott, Architect *i*2000) | Plasma (EDTA) | Abbott TIMP-1 Controls high/medium/low (high:290.86 ng/mL; medium:100.11 ng/mL; low:50.04 ng/mL)* every 24h  Laboratory-internal Serum-Control sample every 24h | 1.91 | 3.13 |
| Troponin I (ng/mL) | TnI | Chemiluminescent microparticle immunoassay CMIA (Abbott, Architect *i*2000) | Serum | Abbott *STAT* Troponin-I Controls low/medium/high (high:16,41 ng/mL; medium:0,61 ng/mL; low:0,16 ng/mL)* every 24h  Laboratory-internal Serum-Control sample every 24h | 2.36 | 4.8 |
| Vitamin B12 (active; Holotranscobalamin) (pmol/L) | active B12 | Microparticle enzyme immunoassay MEIA(Abbott,AxSYM) | Serum | Abbott AxSYM Acitve-B12 Controls high/low (high:48 pmol/L; low:21 pmol/L)* every 24h  Laboratory-internal Serum-Control sample every 24h | 8.17 | 5.33 |
| Vitamin B12 (pg/mL) | B12 | Chemoluminescent microparticle immunoassay CMIA (Abbott, Architect *i*2000) | Serum | Abbott B12 Controls low/medium/high (high:800 pg/mL; medium:400 pg/mL; low:200 pg/mL)* every 24h  Laboratory-internal Serum-Control sample every 24h | 5.94 | 5.57 |
| Determinations that were done at the laboratory of the National Institute of Health and Welfare, Turku, Finland, for the Health 2000 cohort^§^ | | | | | | |
| Apolipoprotein A1 (g/L) | Apo A | Immunoturbidometric assay (Orion Diagnostics, Espoo, Finland, Olympos AU400) | Serum |  |  |  |
| Apolipoprotein B (g/L) | Apo B | Immunoturbidometric assay (Orion Diagnostics, Espoo, Finland, Olympos AU400) | Serum |  |  |  |
| Insulin |  | Microparticle enzyme immunoassay (Abbott Laboratories, Diagnostic Division, Dainabat, Japan) | Serum | Abbott Insulin Controls : low 17.02 µU/mL, high 150.25 µU/mL |  |  |

**Supporting Table S1: Methods for biomarker determinations and quality control results**.

* Concentration of controls lot-dependent, examples are given

^†^ Samples of patients used as controls, not standardized

^‡^ Intra- and inter-assay coefficient of variation were calculated from SD divided by the mean x 100. For intra-assay CVs one laboratory-internal control was used 15 times at minimum within the assay in the same day. For inter-assay CVs the same laboratory-internal control was used each day.

Laboratory-internal serum and plasma controls were pooled serum and plasma samples of known concentration for each biomarker measured.

www.abbottdiagnostics.com, www.biorad.com, www.brahms.de, www.diadexus.com, www.mblintl.com, www.rndsystems.com, www.roche.de/diagnostics, www.sentineldiagnostics.com

^§^All other laboratory determinations were done at the University of Mainz, Germany.
