# Supplementary material for: Thirty-One Novel Biomarkers as Predictors for Clinically Incident Diabetes
Source: PLoS One. 2010 Apr 9;5(4):e10100. doi: 10.1371/journal.pone.0010100 (PMC2852424; doi:10.1371/journal.pone.0010100)
Supplement: Table S2 — Geometric means (inter-quartile range) of biomarkers analyzed in the FINRISK97 and Health 2000 cohorts. (0.10 MB DOC) [file pone.0010100.s003.doc]

|  | **Men^*^** | **Women^*^** | **Total** |
| --- | --- | --- | --- |
|  |  |  |  |
| ***FINRISK97*** | (n=3922) | (n=3905) | (n=7827) |
| Lipid-related markers |  |  |  |
| Apolipoprotein B-100 (g/L) | 1.0 (0.9-1.2) | 0.9 (0.8-1.1) | 1.0 (0.8-1.2) |
| Apolipoprotein A1 (g/L) | 1.4 (1.3-1.6) | 1.6 (1.4-1.8) | 1.5 (1.3-1.7) |
| Phospolipase A_2_ mass (ng/mL) | 254.5 (219.2-298.6) | 241.2 (209.5-279.6) | 247.8 (213.9-288.4) |
| Phospolipase A_2_ activity (mmol/min/ml) | 251.4 (218.8-300.6) | 213.4 (183.3-256.7) | 231.6 (197.8-282.3) |
| Paraoxonase-1 | 92.6 (52.4-171.2) | 91.8 (52.0-170.0) | 92.3 (52.0-170.6) |
|  |  |  |  |
| Metabolic markers |  |  |  |
| Insulin (µU/mL) | 5.5 (3.8-8.0) | 5.0 (3.5-7.2) | 5.3 (3.6-7.6) |
| Adiponectin (ng/mL) | 4268.5 (2886.3-6383.3) | 7363.2 (5103.6-10898.0) | 5602.3 (3645.0-8838.1) |
| Leptin (pg/mL) | 4731.3 (2543.0-8194.2) | 13956.7 (8552.2-23734.0) | 8120.3 (4128.0-16268.0) |
| Placental Growth Factor (pg/mL) | 15.2 (12.9-17.8) | 14.4 (11.9-16.3) | 14.8 (12.3-17.1) |
| D-dimer (ng/mL) | 150.9 (91.0-232.0) | 177.9 (113.0-264.0) | 163.7 (100.0-250.0) |
| Ferritin (ng/mL) | 99.9 (63.3-177.2) | 31.4 (16.3-62.3) | 56.1 (27.6-124.5) |
| Gamma-glutamyl transferase | 34.0 (22.0-47.0) | 20.7 (14.0-26.0) | 26.5 (17.0-37.0) |
| Markers of oxidative stress and antioxidants | | | |
| Vitamin B12 (pg/mL) | 485.6 (388.1-627.0) | 494.4 (384.0-650.0) | 490.0 (386.0-637.0) |
| Active vitamin B12 (pg/mL) | 73.6 (58.2-99.2) | 75.9 (60.0-104.2) | 74.7 (59.1-101.5) |
| Homocysteine (μmol/L) | 12.8 (10.8-14.9) | 10.3 (8.6-12.2) | 11.5 (9.5-13.7) |
| Myeloperoxidase (μg/L) | 15.4 (10.7-20.1) | 14.3 (10.1-19.0) | 14.8 (10.4-19.5) |
| Inflammatory markers | | | |
| C-reactive protein (mg/L) | 1.2 (0.6-2.4) | 1.2 (0.5-2.5) | 1.2 (0.6-2.4) |
| Interleukin-18 (pg/mL) | 264.3 (204.9-335.0) | 212.9 (157.5-272.2) | 237.3 (176.6-307.9) |
| Interleukin-1 receptor antagonist (pg/mL) | 227.2 (167.1-294.5) | 259.4 (185.4-343.4) | 242.6 (175.4-318.8) |
| Neopterin (mmol/L) | 5.9 (4.7-6.9) | 6.0 (4.8-7.0) | 6.0 (4.8-6.9) |
| Renal Function markers | | | |
| Creatinine (mg/dL) | 1.0  (0.9-1.0) | 0.8  (0.7-0.9) | 0.9  (0.8-1.0) |
| Cystatine-C (mg/L) | 0.9  (0.8-0.9) | 0.8  (0.7-0.9) | 0.8  (0.7-0.9) |
| Neurohumoral/ Hemodynamic/ Remodelling Markers | | | |
| Pro atrial natriuretic peptide (µmol/L) | 42.6 (29.6-57.0) | 46.2 (34.0-60.3) | 44.4 (31.9-59.0) |
| NT-Pro B-type natriuretic peptide (µg/mL) | 31.6 (13.6-66.9) | 53.6 (31.9-93.5) | 41.1 (20.8-82.6) |
| B-type natriuretic peptide (pg/mL) | 10.7 (5.5.-23.1) | 15.0 (9.1-29.4) | 12.7 (7.2-27.0) |
| MR-Pro adronomedullin | 0.5 (0.4-0.5) | 0.5 (0.4-0.6) | 0.5 (0.4-0.6) |
| CT-pro arginine vasopressin | 4.8 (2.9-8.0) | 2.7 (1.7-4.3) | 3.6 (2.1-6.1) |
| Pro endothelin-1 (pmol/L) | 50.6 (44.6-59.9) | 51.8 (46.6-62.4) | 51.2 (45.5-61.3) |
| Tissue inhibitor of matrix metalloproteinase (ng/mL) | 90.6 (77.4-102.1) | 84.8 (74.1-95.5) | 87.7 (75.6-98.8) |
| Necrosis | | | |
| Troponin I (ng/mL) | 0.0 (0.0-0.0) | 0.0 (0.0-0.0) | 0.0 (0.0-0.0) |
| Creatine kinase MB (ng/mL) | 1.2 (0.8-1.8) | 1.0 (0.7-1.4) | 1.1 (0.7-1.6) |
|  |  |  |  |
| ***Health 2000*** | (n=2272) | (n=2704) | (n=4976) |
| Lipid-related markers | | | |
| Apolipoprotein B-100 (g/L) | 1.3 (1.1-1.5) | 1.1 (1.0-1.4) | 1.2 (1.0-1.4) |
| Apolipoprotein A1 (g/L) | 1.5 (1.3-1.7) | 1.7 (1.5-1.9) | 1.6 (1.4-1.8) |
| Metabolic markers | | | |
| Insulin (µU/mL) | 7.3 (4.7-10.7) | 6.6 (4.5-9.6) | 6.9 (4.6-10.1) |
| Adiponectin (ng/ml) | 5274.0 (3612.0-7876.0) | 8905.3 (6174.8-13303.0) | 7011.0 (4638.5-11033.0) |
| Leptin (pg/mL) | 5994.0 (3243.0-10692.3) | 18167.2 (11111.8-32054.8) | 10950.0 (5571.1-23036.5) |
| Ferritin (ng/mL) | 108.1  (69.9-184.0) | 36.8 (19.6-73.2) | 60.2 (31.6-126.7) |
| Gamma-glutamyl transferase | 35.1 (22.0-51.0) | 21.7 (15.0-29.0) | 27.0 (17.0-38.0) |
| Markers of oxidative stress and antioxidants | | | |
| Homocysteine (μmol/L) | 12.3 (10.2 -14.2) | 10.4 (8.4-12.5) | 11.2 (9.2-13.4) |
| Inflammatory markers | | | |
| C-reactive protein (mg/L) | 0.7 (0.3-1.8) | 0.7 (0.3-2.0) | 0.7 (0.3-1.9) |
| Interleukin-1 receptor antagonist (pg/mL) | 297.9 (211.0-410.2) | 335.9 (231.4-472.3) | 318.0 (220.9-440.4) |

**Supporting Table S2: Geometric means (inter-quartile range) of biomarkers analyzed in the FINRISK97 and Health 2000 cohorts.**

^*^ After excluding persons with prevalent diabetes at baseline.
