# Supplementary material for: Thirty-One Novel Biomarkers as Predictors for Clinically Incident Diabetes
Source: PLoS One. 2010 Apr 9;5(4):e10100. doi: 10.1371/journal.pone.0010100 (PMC2852424; doi:10.1371/journal.pone.0010100)
Supplement: Table S3 — Rank correlation matrix. FINRISK97, men and women combined. (0.43 MB DOC) [file pone.0010100.s004.doc]

| Variable | AGE1 | SYSTM | DIASTM | HDLA | NONHDL | TRIGLA | BMI | WHR | ACTIVE_B12 | ADIPONECTIN | APO_A | APO_B | APO_ABRATIO | BNP | CK_MB | CREATININE | CRP | CRP_MPC | CT_PRO_AVP | CT_PRO_ET1 | CYSTATIN_C | D_DIMER | FERRITIN | GGT_MPC | GLUCOSE | GLUCOSE_R | HOMOCYSTEINE | IL_18 | IL_1_RA | INSULIN_M | INSULIN_R | LEPTIN | MPO | MR_PRO_ADM | MR_PRO_ANP | NEOPTERIN | NT_PRO_BNP | PLA_A | PLA_M | PLGF | PON_1 | TIMP_1 | VIT_B12 | TROPONIN |
| --- | --- | --- | --- | --- | --- | --- | --- | --- | --- | --- | --- | --- | --- | --- | --- | --- | --- | --- | --- | --- | --- | --- | --- | --- | --- | --- | --- | --- | --- | --- | --- | --- | --- | --- | --- | --- | --- | --- | --- | --- | --- | --- | --- | --- |
| AGE1 | 1.00 | 0.47 | 0.32 | -0.07 | 0.37 | 0.25 | 0.32 | 0.37 | -0.06 | 0.08 | 0.03 | 0.33 | -0.27 | 0.43 | 0.16 | 0.13 | 0.25 | 0.25 | 0.13 | 0.36 | 0.49 | 0.21 | 0.19 | 0.23 | 0.29 | 0.29 | 0.35 | 0.20 | 0.07 | 0.17 | 0.18 | 0.16 | 0.06 | 0.54 | 0.56 | 0.32 | 0.41 | 0.14 | 0.12 | 0.40 | -0.10 | 0.34 | -0.06 | 0.31 |
| SYSTM | 0.47 | 1.00 | 0.65 | -0.11 | 0.29 | 0.27 | 0.35 | 0.37 | -0.02 | -0.06 | 0.00 | 0.27 | -0.22 | 0.18 | 0.15 | 0.14 | 0.19 | 0.19 | 0.13 | 0.18 | 0.28 | 0.08 | 0.22 | 0.31 | 0.24 | 0.26 | 0.26 | 0.17 | 0.08 | 0.23 | 0.24 | 0.07 | 0.09 | 0.29 | 0.26 | 0.10 | 0.19 | 0.14 | 0.08 | 0.20 | 0.01 | 0.27 | -0.02 | 0.20 |
| DIASTM | 0.32 | 0.65 | 1.00 | -0.14 | 0.28 | 0.29 | 0.36 | 0.38 | -0.02 | -0.07 | -0.02 | 0.28 | -0.24 | 0.03 | 0.12 | 0.14 | 0.16 | 0.16 | 0.12 | 0.14 | 0.23 | 0.03 | 0.22 | 0.33 | 0.15 | 0.16 | 0.20 | 0.18 | 0.13 | 0.21 | 0.23 | 0.09 | 0.10 | 0.22 | 0.12 | 0.07 | 0.02 | 0.11 | 0.08 | 0.13 | 0.04 | 0.21 | 0.00 | 0.10 |
| HDLA | -0.07 | -0.11 | -0.14 | 1.00 | -0.24 | -0.48 | -0.32 | -0.47 | 0.02 | 0.38 | 0.80 | -0.28 | 0.68 | 0.07 | 0.02 | -0.25 | -0.18 | -0.18 | -0.18 | -0.06 | -0.29 | -0.01 | -0.28 | -0.25 | -0.08 | -0.08 | -0.22 | -0.30 | -0.22 | -0.32 | -0.32 | 0.05 | -0.09 | -0.08 | 0.06 | -0.10 | 0.10 | -0.35 | -0.05 | -0.19 | 0.05 | -0.15 | -0.01 | -0.06 |
| NONHDL | 0.37 | 0.29 | 0.28 | -0.24 | 1.00 | 0.56 | 0.35 | 0.37 | 0.05 | -0.12 | -0.05 | 0.91 | -0.76 | 0.00 | 0.06 | 0.15 | 0.21 | 0.21 | 0.12 | 0.13 | 0.28 | 0.06 | 0.23 | 0.32 | 0.13 | 0.13 | 0.23 | 0.19 | 0.15 | 0.24 | 0.25 | 0.11 | 0.07 | 0.25 | 0.10 | 0.05 | -0.02 | 0.42 | 0.15 | 0.19 | 0.04 | 0.23 | 0.03 | 0.08 |
| TRIGLA | 0.25 | 0.27 | 0.29 | -0.48 | 0.56 | 1.00 | 0.42 | 0.49 | -0.03 | -0.26 | -0.18 | 0.55 | -0.55 | 0.00 | 0.03 | 0.22 | 0.27 | 0.28 | 0.18 | 0.13 | 0.34 | 0.05 | 0.28 | 0.40 | 0.15 | 0.15 | 0.24 | 0.28 | 0.26 | 0.47 | 0.46 | 0.14 | 0.10 | 0.27 | 0.06 | 0.05 | -0.03 | 0.25 | 0.03 | 0.30 | 0.04 | 0.23 | -0.03 | 0.09 |
| BMI | 0.32 | 0.35 | 0.36 | -0.32 | 0.35 | 0.42 | 1.00 | 0.55 | 0.03 | -0.17 | -0.17 | 0.36 | -0.39 | 0.07 | 0.14 | 0.13 | 0.41 | 0.42 | 0.16 | 0.20 | 0.34 | 0.13 | 0.25 | 0.37 | 0.12 | 0.12 | 0.19 | 0.26 | 0.39 | 0.48 | 0.49 | 0.41 | 0.09 | 0.40 | 0.12 | 0.10 | 0.03 | 0.14 | 0.05 | 0.26 | 0.03 | 0.24 | 0.01 | 0.12 |
| WHR | 0.37 | 0.37 | 0.38 | -0.47 | 0.37 | 0.49 | 0.55 | 1.00 | -0.02 | -0.35 | -0.28 | 0.39 | -0.48 | -0.03 | 0.18 | 0.39 | 0.29 | 0.30 | 0.35 | 0.11 | 0.41 | 0.03 | 0.53 | 0.56 | 0.22 | 0.22 | 0.40 | 0.38 | 0.17 | 0.36 | 0.37 | -0.10 | 0.11 | 0.27 | 0.05 | 0.07 | -0.10 | 0.31 | 0.12 | 0.33 | 0.01 | 0.30 | -0.01 | 0.19 |
| ACTIVE_B12 | -0.06 | -0.02 | -0.02 | 0.02 | 0.05 | -0.03 | 0.03 | -0.02 | 1.00 | 0.01 | -0.02 | 0.03 | -0.03 | -0.10 | 0.08 | 0.01 | -0.01 | -0.01 | -0.07 | 0.00 | -0.02 | -0.02 | 0.03 | 0.03 | -0.05 | -0.05 | -0.16 | -0.02 | -0.01 | -0.01 | -0.02 | 0.03 | -0.02 | -0.05 | -0.06 | -0.03 | -0.10 | 0.00 | -0.02 | -0.08 | 0.04 | 0.03 | 0.65 | -0.05 |
| ADIPONECTIN | 0.08 | -0.06 | -0.07 | 0.38 | -0.12 | -0.26 | -0.17 | -0.35 | 0.01 | 1.00 | 0.30 | -0.14 | 0.28 | 0.20 | -0.03 | -0.26 | -0.08 | -0.08 | -0.16 | 0.07 | -0.13 | 0.07 | -0.23 | -0.22 | -0.04 | -0.04 | -0.10 | -0.16 | -0.10 | -0.19 | -0.19 | 0.22 | -0.04 | 0.05 | 0.18 | 0.08 | 0.25 | -0.18 | -0.01 | -0.06 | -0.03 | -0.05 | 0.02 | -0.01 |
| APO_A | 0.03 | 0.00 | -0.02 | 0.80 | -0.05 | -0.18 | -0.17 | -0.28 | -0.02 | 0.30 | 1.00 | -0.02 | 0.55 | 0.08 | 0.08 | -0.16 | -0.06 | -0.08 | -0.10 | -0.02 | -0.16 | 0.01 | -0.14 | -0.10 | -0.09 | -0.09 | -0.16 | -0.16 | -0.14 | -0.13 | -0.13 | 0.09 | -0.05 | 0.01 | 0.09 | -0.09 | 0.09 | -0.24 | 0.00 | -0.06 | 0.09 | -0.06 | 0.03 | -0.04 |
| APO_B | 0.33 | 0.27 | 0.28 | -0.28 | 0.91 | 0.55 | 0.36 | 0.39 | 0.03 | -0.14 | -0.02 | 1.00 | -0.81 | 0.01 | 0.08 | 0.15 | 0.27 | 0.24 | 0.13 | 0.12 | 0.29 | 0.06 | 0.27 | 0.34 | 0.08 | 0.09 | 0.21 | 0.23 | 0.18 | 0.28 | 0.29 | 0.11 | 0.09 | 0.25 | 0.09 | 0.06 | -0.02 | 0.41 | 0.10 | 0.22 | 0.05 | 0.21 | 0.05 | 0.07 |
| APO_ABRATIO | -0.27 | -0.22 | -0.24 | 0.68 | -0.76 | -0.55 | -0.39 | -0.48 | -0.03 | 0.28 | 0.55 | -0.81 | 1.00 | 0.03 | -0.02 | -0.21 | -0.25 | -0.25 | -0.17 | -0.12 | -0.33 | -0.05 | -0.30 | -0.34 | -0.12 | -0.12 | -0.27 | -0.29 | -0.22 | -0.31 | -0.31 | -0.04 | -0.10 | -0.20 | -0.02 | -0.11 | 0.07 | -0.47 | -0.08 | -0.21 | 0.01 | -0.21 | -0.03 | -0.09 |
| BNP | 0.43 | 0.18 | 0.03 | 0.07 | 0.00 | 0.00 | 0.07 | -0.03 | -0.10 | 0.20 | 0.08 | 0.01 | 0.03 | 1.00 | 0.05 | -0.09 | 0.14 | 0.14 | 0.00 | 0.31 | 0.20 | 0.19 | -0.08 | -0.04 | 0.09 | 0.07 | 0.07 | 0.01 | 0.05 | -0.01 | 0.00 | 0.18 | 0.04 | 0.38 | 0.70 | 0.21 | 0.82 | -0.07 | -0.02 | 0.26 | -0.06 | 0.12 | -0.07 | 0.21 |
| CK_MB | 0.16 | 0.15 | 0.12 | 0.02 | 0.06 | 0.03 | 0.14 | 0.18 | 0.08 | -0.03 | 0.08 | 0.08 | -0.02 | 0.05 | 1.00 | 0.11 | 0.01 | 0.00 | 0.07 | 0.10 | 0.07 | 0.01 | 0.10 | 0.08 | 0.01 | 0.02 | 0.04 | 0.07 | -0.04 | -0.02 | -0.01 | -0.14 | -0.04 | 0.04 | 0.10 | 0.04 | 0.02 | 0.00 | -0.01 | -0.01 | 0.04 | 0.12 | 0.07 | 0.14 |
| CREATININE | 0.13 | 0.14 | 0.14 | -0.25 | 0.15 | 0.22 | 0.13 | 0.39 | 0.01 | -0.26 | -0.16 | 0.15 | -0.21 | -0.09 | 0.11 | 1.00 | 0.03 | 0.03 | 0.26 | 0.06 | 0.40 | -0.05 | 0.31 | 0.27 | 0.10 | 0.11 | 0.36 | 0.18 | -0.04 | 0.13 | 0.13 | -0.24 | 0.04 | 0.12 | 0.03 | 0.12 | -0.13 | 0.22 | 0.08 | 0.11 | 0.01 | 0.19 | 0.01 | 0.14 |
| CRP | 0.25 | 0.19 | 0.16 | -0.18 | 0.21 | 0.27 | 0.41 | 0.29 | -0.01 | -0.08 | -0.06 | 0.27 | -0.25 | 0.14 | 0.01 | 0.03 | 1.00 | 0.97 | 0.15 | 0.19 | 0.30 | 0.21 | 0.19 | 0.28 | 0.04 | 0.04 | 0.14 | 0.26 | 0.40 | 0.32 | 0.33 | 0.27 | 0.17 | 0.36 | 0.14 | 0.25 | 0.17 | 0.03 | 0.04 | 0.31 | -0.04 | 0.25 | 0.02 | 0.12 |
| CRP_MPC | 0.25 | 0.19 | 0.16 | -0.18 | 0.21 | 0.28 | 0.42 | 0.30 | -0.01 | -0.08 | -0.08 | 0.24 | -0.25 | 0.14 | 0.00 | 0.03 | 0.97 | 1.00 | 0.15 | 0.19 | 0.31 | 0.21 | 0.17 | 0.29 | 0.06 | 0.05 | 0.14 | 0.25 | 0.41 | 0.31 | 0.31 | 0.27 | 0.17 | 0.37 | 0.14 | 0.26 | 0.18 | 0.04 | 0.04 | 0.32 | -0.04 | 0.26 | 0.00 | 0.11 |
| CT_PRO_AVP | 0.13 | 0.13 | 0.12 | -0.18 | 0.12 | 0.18 | 0.16 | 0.35 | -0.07 | -0.16 | -0.10 | 0.13 | -0.17 | 0.00 | 0.07 | 0.26 | 0.15 | 0.15 | 1.00 | 0.10 | 0.29 | 0.06 | 0.29 | 0.25 | 0.11 | 0.11 | 0.24 | 0.21 | 0.11 | 0.17 | 0.18 | -0.11 | 0.15 | 0.17 | 0.03 | 0.10 | -0.03 | 0.17 | 0.07 | 0.16 | 0.00 | 0.18 | -0.04 | 0.12 |
| CT_PRO_ET1 | 0.36 | 0.18 | 0.14 | -0.06 | 0.13 | 0.13 | 0.20 | 0.11 | 0.00 | 0.07 | -0.02 | 0.12 | -0.12 | 0.31 | 0.10 | 0.06 | 0.19 | 0.19 | 0.10 | 1.00 | 0.40 | 0.13 | -0.02 | 0.09 | 0.05 | 0.10 | 0.22 | 0.12 | 0.15 | 0.09 | 0.11 | 0.19 | 0.09 | 0.49 | 0.32 | 0.31 | 0.26 | 0.00 | 0.05 | 0.10 | -0.06 | 0.22 | 0.01 | 0.17 |
| CYSTATIN_C | 0.49 | 0.28 | 0.23 | -0.29 | 0.28 | 0.34 | 0.34 | 0.41 | -0.02 | -0.13 | -0.16 | 0.29 | -0.33 | 0.20 | 0.07 | 0.40 | 0.30 | 0.31 | 0.29 | 0.40 | 1.00 | 0.15 | 0.26 | 0.29 | 0.15 | 0.15 | 0.45 | 0.33 | 0.22 | 0.26 | 0.27 | 0.09 | 0.22 | 0.51 | 0.31 | 0.38 | 0.18 | 0.23 | 0.13 | 0.41 | -0.07 | 0.45 | -0.05 | 0.24 |
| D_DIMER | 0.21 | 0.08 | 0.03 | -0.01 | 0.06 | 0.05 | 0.13 | 0.03 | -0.02 | 0.07 | 0.01 | 0.06 | -0.05 | 0.19 | 0.01 | -0.05 | 0.21 | 0.21 | 0.06 | 0.13 | 0.15 | 1.00 | -0.05 | 0.02 | 0.03 | 0.03 | 0.05 | 0.09 | 0.14 | 0.09 | 0.09 | 0.18 | 0.15 | 0.14 | 0.20 | 0.21 | 0.22 | -0.01 | -0.05 | 0.19 | -0.03 | 0.18 | 0.02 | 0.08 |
| FERRITIN | 0.19 | 0.22 | 0.22 | -0.28 | 0.23 | 0.28 | 0.25 | 0.53 | 0.03 | -0.23 | -0.14 | 0.27 | -0.30 | -0.08 | 0.10 | 0.31 | 0.19 | 0.17 | 0.29 | -0.02 | 0.26 | -0.05 | 1.00 | 0.47 | 0.17 | 0.17 | 0.26 | 0.27 | 0.06 | 0.17 | 0.18 | -0.18 | 0.10 | 0.10 | -0.02 | -0.02 | -0.11 | 0.26 | 0.19 | 0.25 | 0.02 | 0.15 | 0.04 | 0.12 |
| GGT_MPC | 0.23 | 0.31 | 0.33 | -0.25 | 0.32 | 0.40 | 0.37 | 0.56 | 0.03 | -0.22 | -0.10 | 0.34 | -0.34 | -0.04 | 0.08 | 0.27 | 0.28 | 0.29 | 0.25 | 0.09 | 0.29 | 0.02 | 0.47 | 1.00 | 0.19 | 0.20 | 0.29 | 0.30 | 0.18 | 0.30 | 0.31 | -0.02 | 0.12 | 0.21 | 0.02 | 0.01 | -0.09 | 0.23 | 0.09 | 0.25 | 0.03 | 0.25 | 0.06 | 0.13 |
| GLUCOSE | 0.29 | 0.24 | 0.15 | -0.08 | 0.13 | 0.15 | 0.12 | 0.22 | -0.05 | -0.04 | -0.09 | 0.08 | -0.12 | 0.09 | 0.01 | 0.10 | 0.04 | 0.06 | 0.11 | 0.05 | 0.15 | 0.03 | 0.17 | 0.19 | 1.00 | 0.96 | 0.12 | 0.08 | 0.01 | 0.26 | 0.24 | 0.00 | 0.02 | 0.12 | 0.14 | 0.03 | 0.10 | 0.11 | 0.05 | 0.20 | -0.02 | 0.14 | -0.06 | 0.11 |
| GLUCOSE_R | 0.29 | 0.26 | 0.16 | -0.08 | 0.13 | 0.15 | 0.12 | 0.22 | -0.05 | -0.04 | -0.09 | 0.09 | -0.12 | 0.07 | 0.02 | 0.11 | 0.04 | 0.05 | 0.11 | 0.10 | 0.15 | 0.03 | 0.17 | 0.20 | 0.96 | 1.00 | 0.13 | 0.07 | 0.01 | 0.23 | 0.25 | 0.01 | 0.03 | 0.13 | 0.13 | 0.05 | 0.09 | 0.10 | 0.05 | 0.14 | -0.02 | 0.14 | -0.06 | 0.11 |
| HOMOCYSTEINE | 0.35 | 0.26 | 0.20 | -0.22 | 0.23 | 0.24 | 0.19 | 0.40 | -0.16 | -0.10 | -0.16 | 0.21 | -0.27 | 0.07 | 0.04 | 0.36 | 0.14 | 0.14 | 0.24 | 0.22 | 0.45 | 0.05 | 0.26 | 0.29 | 0.12 | 0.13 | 1.00 | 0.25 | 0.04 | 0.17 | 0.17 | -0.10 | 0.13 | 0.28 | 0.16 | 0.20 | 0.06 | 0.20 | 0.13 | 0.22 | -0.03 | 0.25 | -0.18 | 0.19 |
| IL_18 | 0.20 | 0.17 | 0.18 | -0.30 | 0.19 | 0.28 | 0.26 | 0.38 | -0.02 | -0.16 | -0.16 | 0.23 | -0.29 | 0.01 | 0.07 | 0.18 | 0.26 | 0.25 | 0.21 | 0.12 | 0.33 | 0.09 | 0.27 | 0.30 | 0.08 | 0.07 | 0.25 | 1.00 | 0.22 | 0.22 | 0.23 | 0.00 | 0.17 | 0.20 | 0.04 | 0.20 | -0.01 | 0.19 | 0.09 | 0.25 | -0.02 | 0.22 | 0.02 | 0.10 |
| IL_1_RA | 0.07 | 0.08 | 0.13 | -0.22 | 0.15 | 0.26 | 0.39 | 0.17 | -0.01 | -0.10 | -0.14 | 0.18 | -0.22 | 0.05 | -0.04 | -0.04 | 0.40 | 0.41 | 0.11 | 0.15 | 0.22 | 0.14 | 0.06 | 0.18 | 0.01 | 0.01 | 0.04 | 0.22 | 1.00 | 0.30 | 0.31 | 0.35 | 0.20 | 0.29 | 0.04 | 0.18 | 0.08 | 0.01 | -0.01 | 0.19 | -0.01 | 0.17 | 0.01 | 0.01 |
| INSULIN_M | 0.17 | 0.23 | 0.21 | -0.32 | 0.24 | 0.47 | 0.48 | 0.36 | -0.01 | -0.19 | -0.13 | 0.28 | -0.31 | -0.01 | -0.02 | 0.13 | 0.32 | 0.31 | 0.17 | 0.09 | 0.26 | 0.09 | 0.17 | 0.30 | 0.26 | 0.23 | 0.17 | 0.22 | 0.30 | 1.00 | 0.98 | 0.33 | 0.11 | 0.26 | 0.02 | 0.07 | -0.03 | 0.13 | 0.02 | 0.30 | 0.00 | 0.21 | 0.01 | 0.11 |
| INSULIN_R | 0.18 | 0.24 | 0.23 | -0.32 | 0.25 | 0.46 | 0.49 | 0.37 | -0.02 | -0.19 | -0.13 | 0.29 | -0.31 | 0.00 | -0.01 | 0.13 | 0.33 | 0.31 | 0.18 | 0.11 | 0.27 | 0.09 | 0.18 | 0.31 | 0.24 | 0.25 | 0.17 | 0.23 | 0.31 | 0.98 | 1.00 | 0.34 | 0.11 | 0.27 | 0.03 | 0.08 | -0.02 | 0.13 | 0.02 | 0.29 | 0.00 | 0.21 | 0.01 | 0.11 |
| LEPTIN | 0.16 | 0.07 | 0.09 | 0.05 | 0.11 | 0.14 | 0.41 | -0.10 | 0.03 | 0.22 | 0.09 | 0.11 | -0.04 | 0.18 | -0.14 | -0.24 | 0.27 | 0.27 | -0.11 | 0.19 | 0.09 | 0.18 | -0.18 | -0.02 | 0.00 | 0.01 | -0.10 | 0.00 | 0.35 | 0.33 | 0.34 | 1.00 | 0.06 | 0.33 | 0.18 | 0.10 | 0.22 | -0.11 | -0.05 | 0.12 | -0.01 | 0.08 | 0.01 | -0.01 |
| MPO | 0.06 | 0.09 | 0.10 | -0.09 | 0.07 | 0.10 | 0.09 | 0.11 | -0.02 | -0.04 | -0.05 | 0.09 | -0.10 | 0.04 | -0.04 | 0.04 | 0.17 | 0.17 | 0.15 | 0.09 | 0.22 | 0.15 | 0.10 | 0.12 | 0.02 | 0.03 | 0.13 | 0.17 | 0.20 | 0.11 | 0.11 | 0.06 | 1.00 | 0.08 | 0.02 | 0.16 | 0.03 | 0.08 | 0.08 | 0.13 | -0.02 | 0.15 | 0.05 | 0.05 |
| MR_PRO_ADM | 0.54 | 0.29 | 0.22 | -0.08 | 0.25 | 0.27 | 0.40 | 0.27 | -0.05 | 0.05 | 0.01 | 0.25 | -0.20 | 0.38 | 0.04 | 0.12 | 0.36 | 0.37 | 0.17 | 0.49 | 0.51 | 0.14 | 0.10 | 0.21 | 0.12 | 0.13 | 0.28 | 0.20 | 0.29 | 0.26 | 0.27 | 0.33 | 0.08 | 1.00 | 0.42 | 0.32 | 0.35 | 0.05 | 0.06 | 0.34 | -0.05 | 0.28 | -0.09 | 0.22 |
| MR_PRO_ANP | 0.56 | 0.26 | 0.12 | 0.06 | 0.10 | 0.06 | 0.12 | 0.05 | -0.06 | 0.18 | 0.09 | 0.09 | -0.02 | 0.70 | 0.10 | 0.03 | 0.14 | 0.14 | 0.03 | 0.32 | 0.31 | 0.20 | -0.02 | 0.02 | 0.14 | 0.13 | 0.16 | 0.04 | 0.04 | 0.02 | 0.03 | 0.18 | 0.02 | 0.42 | 1.00 | 0.25 | 0.68 | -0.02 | 0.01 | 0.29 | -0.06 | 0.22 | -0.06 | 0.25 |
| NEOPTERIN | 0.32 | 0.10 | 0.07 | -0.10 | 0.05 | 0.05 | 0.10 | 0.07 | -0.03 | 0.08 | -0.09 | 0.06 | -0.11 | 0.21 | 0.04 | 0.12 | 0.25 | 0.26 | 0.10 | 0.31 | 0.38 | 0.21 | -0.02 | 0.01 | 0.03 | 0.05 | 0.20 | 0.20 | 0.18 | 0.07 | 0.08 | 0.10 | 0.16 | 0.32 | 0.25 | 1.00 | 0.24 | 0.01 | 0.00 | 0.18 | -0.09 | 0.26 | -0.03 | 0.15 |
| NT_PRO_BNP | 0.41 | 0.19 | 0.02 | 0.10 | -0.02 | -0.03 | 0.03 | -0.10 | -0.10 | 0.25 | 0.09 | -0.02 | 0.07 | 0.82 | 0.02 | -0.13 | 0.17 | 0.18 | -0.03 | 0.26 | 0.18 | 0.22 | -0.11 | -0.09 | 0.10 | 0.09 | 0.06 | -0.01 | 0.08 | -0.03 | -0.02 | 0.22 | 0.03 | 0.35 | 0.68 | 0.24 | 1.00 | -0.08 | 0.00 | 0.22 | -0.08 | 0.15 | -0.08 | 0.21 |
| PLA_A | 0.14 | 0.14 | 0.11 | -0.35 | 0.42 | 0.25 | 0.14 | 0.31 | 0.00 | -0.18 | -0.24 | 0.41 | -0.47 | -0.07 | 0.00 | 0.22 | 0.03 | 0.04 | 0.17 | 0.00 | 0.23 | -0.01 | 0.26 | 0.23 | 0.11 | 0.10 | 0.20 | 0.19 | 0.01 | 0.13 | 0.13 | -0.11 | 0.08 | 0.05 | -0.02 | 0.01 | -0.08 | 1.00 | 0.28 | 0.13 | 0.00 | 0.13 | 0.02 | 0.04 |
| PLA_M | 0.12 | 0.08 | 0.08 | -0.05 | 0.15 | 0.03 | 0.05 | 0.12 | -0.02 | -0.01 | 0.00 | 0.10 | -0.08 | -0.02 | -0.01 | 0.08 | 0.04 | 0.04 | 0.07 | 0.05 | 0.13 | -0.05 | 0.19 | 0.09 | 0.05 | 0.05 | 0.13 | 0.09 | -0.01 | 0.02 | 0.02 | -0.05 | 0.08 | 0.06 | 0.01 | 0.00 | 0.00 | 0.28 | 1.00 | 0.02 | -0.05 | 0.06 | 0.02 | 0.03 |
| PLGF | 0.40 | 0.20 | 0.13 | -0.19 | 0.19 | 0.30 | 0.26 | 0.33 | -0.08 | -0.06 | -0.06 | 0.22 | -0.21 | 0.26 | -0.01 | 0.11 | 0.31 | 0.32 | 0.16 | 0.10 | 0.41 | 0.19 | 0.25 | 0.25 | 0.20 | 0.14 | 0.22 | 0.25 | 0.19 | 0.30 | 0.29 | 0.12 | 0.13 | 0.34 | 0.29 | 0.18 | 0.22 | 0.13 | 0.02 | 1.00 | -0.04 | 0.30 | -0.07 | 0.20 |
| PON_1 | -0.10 | 0.01 | 0.04 | 0.05 | 0.04 | 0.04 | 0.03 | 0.01 | 0.04 | -0.03 | 0.09 | 0.05 | 0.01 | -0.06 | 0.04 | 0.01 | -0.04 | -0.04 | 0.00 | -0.06 | -0.07 | -0.03 | 0.02 | 0.03 | -0.02 | -0.02 | -0.03 | -0.02 | -0.01 | 0.00 | 0.00 | -0.01 | -0.02 | -0.05 | -0.06 | -0.09 | -0.08 | 0.00 | -0.05 | -0.04 | 1.00 | -0.02 | 0.05 | -0.08 |
| TIMP_1 | 0.34 | 0.27 | 0.21 | -0.15 | 0.23 | 0.23 | 0.24 | 0.30 | 0.03 | -0.05 | -0.06 | 0.21 | -0.21 | 0.12 | 0.12 | 0.19 | 0.25 | 0.26 | 0.18 | 0.22 | 0.45 | 0.18 | 0.15 | 0.25 | 0.14 | 0.14 | 0.25 | 0.22 | 0.17 | 0.21 | 0.21 | 0.08 | 0.15 | 0.28 | 0.22 | 0.26 | 0.15 | 0.13 | 0.06 | 0.30 | -0.02 | 1.00 | 0.05 | 0.13 |
| VIT_B12 | -0.06 | -0.02 | 0.00 | -0.01 | 0.03 | -0.03 | 0.01 | -0.01 | 0.65 | 0.02 | 0.03 | 0.05 | -0.03 | -0.07 | 0.07 | 0.01 | 0.02 | 0.00 | -0.04 | 0.01 | -0.05 | 0.02 | 0.04 | 0.06 | -0.06 | -0.06 | -0.18 | 0.02 | 0.01 | 0.01 | 0.01 | 0.01 | 0.05 | -0.09 | -0.06 | -0.03 | -0.08 | 0.02 | 0.02 | -0.07 | 0.05 | 0.05 | 1.00 | -0.05 |
| TROPONIN | 0.31 | 0.20 | 0.10 | -0.06 | 0.08 | 0.09 | 0.12 | 0.19 | -0.05 | -0.01 | -0.04 | 0.07 | -0.09 | 0.21 | 0.14 | 0.14 | 0.12 | 0.11 | 0.12 | 0.17 | 0.24 | 0.08 | 0.12 | 0.13 | 0.11 | 0.11 | 0.19 | 0.10 | 0.01 | 0.11 | 0.11 | -0.01 | 0.05 | 0.22 | 0.25 | 0.15 | 0.21 | 0.04 | 0.03 | 0.20 | -0.08 | 0.13 | -0.05 | 1.00 |

**Supporting Table S3: Rank correlation matrix. FINRISK97, men and women combined.**
