# Supplementary material for: Thirty-One Novel Biomarkers as Predictors for Clinically Incident Diabetes
Source: PLoS One. 2010 Apr 9;5(4):e10100. doi: 10.1371/journal.pone.0010100 (PMC2852424; doi:10.1371/journal.pone.0010100)
Supplement: Table S4 — Rank correlation matrix. Health 2000, men and women combined. (0.14 MB DOC) [file pone.0010100.s005.doc]

| Variable | AGE1 | SYSTM | DIASTM | HDLA | NON HDL | TRIGLA | BMI | WHR | ADIPONECTIN | APO _A | APO _B | APO_ABRATIO | CRP | FERRITIN | GGT | GLUCOSE | GLUCOSE_R | HBA1C | HOMOCYSTEINE | IL_1_RA | INSULIN_M | INSULIN_R | LEPTIN |
| --- | --- | --- | --- | --- | --- | --- | --- | --- | --- | --- | --- | --- | --- | --- | --- | --- | --- | --- | --- | --- | --- | --- | --- |
| AGE1 | 1.00 | 0.48 | 0.08 | -0.02 | 0.19 | 0.14 | 0.18 | 0.09 | 0.20 | 0.04 | 0.19 | -0.12 | 0.20 | 0.08 | 0.04 | 0.29 | 0.28 | 0.40 | 0.36 | 0.06 | 0.14 | 0.14 | 0.16 |
| SYSTM | 0.48 | 1.00 | 0.61 | -0.09 | 0.21 | 0.22 | 0.30 | 0.22 | 0.02 | 0.04 | 0.23 | -0.16 | 0.17 | 0.11 | 0.19 | 0.29 | 0.30 | 0.30 | 0.25 | 0.09 | 0.26 | 0.27 | 0.15 |
| DIASTM | 0.08 | 0.61 | 1.00 | -0.14 | 0.21 | 0.24 | 0.33 | 0.31 | -0.12 | 0.00 | 0.24 | -0.19 | 0.11 | 0.17 | 0.26 | 0.18 | 0.19 | 0.18 | 0.13 | 0.10 | 0.25 | 0.26 | 0.09 |
| HDLA | -0.02 | -0.09 | -0.14 | 1.00 | -0.25 | -0.54 | -0.35 | -0.44 | 0.48 | 0.82 | -0.40 | 0.80 | -0.21 | -0.22 | -0.21 | -0.13 | -0.12 | -0.27 | -0.19 | -0.18 | -0.39 | -0.39 | -0.01 |
| NONHDL | 0.19 | 0.21 | 0.21 | -0.25 | 1.00 | 0.51 | 0.25 | 0.26 | -0.12 | 0.04 | 0.93 | -0.68 | 0.14 | 0.19 | 0.24 | 0.18 | 0.18 | 0.28 | 0.15 | 0.05 | 0.22 | 0.23 | 0.08 |
| TRIGLA | 0.14 | 0.22 | 0.24 | -0.54 | 0.51 | 1.00 | 0.38 | 0.39 | -0.31 | -0.14 | 0.67 | -0.60 | 0.23 | 0.25 | 0.33 | 0.18 | 0.18 | 0.32 | 0.20 | 0.21 | 0.43 | 0.43 | 0.17 |
| BMI | 0.18 | 0.30 | 0.33 | -0.35 | 0.25 | 0.38 | 1.00 | 0.44 | -0.22 | -0.18 | 0.32 | -0.36 | 0.38 | 0.21 | 0.31 | 0.21 | 0.21 | 0.33 | 0.14 | 0.31 | 0.56 | 0.57 | 0.52 |
| WHR | 0.09 | 0.22 | 0.31 | -0.44 | 0.26 | 0.39 | 0.44 | 1.00 | -0.42 | -0.28 | 0.36 | -0.45 | 0.22 | 0.48 | 0.47 | 0.24 | 0.25 | 0.35 | 0.30 | 0.14 | 0.37 | 0.38 | -0.16 |
| ADIPONECTIN | 0.20 | 0.02 | -0.12 | 0.48 | -0.12 | -0.31 | -0.22 | -0.42 | 1.00 | 0.38 | -0.23 | 0.40 | -0.09 | -0.25 | -0.24 | -0.08 | -0.08 | -0.19 | -0.04 | -0.09 | -0.28 | -0.28 | 0.11 |
| APO_A | 0.04 | 0.04 | 0.00 | 0.82 | 0.04 | -0.14 | -0.18 | -0.28 | 0.38 | 1.00 | -0.06 | 0.62 | -0.08 | -0.12 | -0.04 | 0.00 | 0.00 | -0.14 | -0.11 | -0.08 | -0.19 | -0.19 | 0.09 |
| APO_B | 0.19 | 0.23 | 0.24 | -0.40 | 0.93 | 0.67 | 0.32 | 0.36 | -0.23 | -0.06 | 1.00 | -0.79 | 0.20 | 0.24 | 0.30 | 0.19 | 0.19 | 0.33 | 0.18 | 0.12 | 0.31 | 0.32 | 0.10 |
| APO_ABRATIO | -0.12 | -0.16 | -0.19 | 0.80 | -0.68 | -0.60 | -0.36 | -0.45 | 0.40 | 0.62 | -0.79 | 1.00 | -0.21 | -0.26 | -0.26 | -0.15 | -0.15 | -0.34 | -0.21 | -0.15 | -0.36 | -0.36 | -0.02 |
| CRP | 0.20 | 0.17 | 0.11 | -0.21 | 0.14 | 0.23 | 0.38 | 0.22 | -0.09 | -0.08 | 0.20 | -0.21 | 1.00 | 0.15 | 0.26 | 0.09 | 0.09 | 0.22 | 0.14 | 0.32 | 0.29 | 0.29 | 0.29 |
| FERRITIN | 0.08 | 0.11 | 0.17 | -0.22 | 0.19 | 0.25 | 0.21 | 0.48 | -0.25 | -0.12 | 0.24 | -0.26 | 0.15 | 1.00 | 0.44 | 0.22 | 0.22 | 0.20 | 0.19 | 0.05 | 0.18 | 0.18 | -0.18 |
| GGT | 0.04 | 0.19 | 0.26 | -0.21 | 0.24 | 0.33 | 0.31 | 0.47 | -0.24 | -0.04 | 0.30 | -0.26 | 0.26 | 0.44 | 1.00 | 0.21 | 0.21 | 0.24 | 0.16 | 0.12 | 0.32 | 0.32 | -0.02 |
| GLUCOSE | 0.29 | 0.29 | 0.18 | -0.13 | 0.18 | 0.18 | 0.21 | 0.24 | -0.08 | 0.00 | 0.19 | -0.15 | 0.09 | 0.22 | 0.21 | 1.00 | 0.94 | 0.41 | 0.18 | 0.05 | 0.37 | 0.35 | 0.04 |
| GLUCOSE_R | 0.28 | 0.30 | 0.19 | -0.12 | 0.18 | 0.18 | 0.21 | 0.25 | -0.08 | 0.00 | 0.19 | -0.15 | 0.09 | 0.22 | 0.21 | 0.94 | 1.00 | 0.40 | 0.19 | 0.06 | 0.34 | 0.38 | 0.04 |
| HBA1C | 0.40 | 0.30 | 0.18 | -0.27 | 0.28 | 0.32 | 0.33 | 0.35 | -0.19 | -0.14 | 0.33 | -0.34 | 0.22 | 0.20 | 0.24 | 0.41 | 0.40 | 1.00 | 0.24 | 0.13 | 0.34 | 0.34 | 0.09 |
| HOMOCYSTEINE | 0.36 | 0.25 | 0.13 | -0.19 | 0.15 | 0.20 | 0.14 | 0.30 | -0.04 | -0.11 | 0.18 | -0.21 | 0.14 | 0.19 | 0.16 | 0.18 | 0.19 | 0.24 | 1.00 | 0.07 | 0.15 | 0.16 | -0.08 |
| IL_1_RA | 0.06 | 0.09 | 0.10 | -0.18 | 0.05 | 0.21 | 0.31 | 0.14 | -0.09 | -0.08 | 0.12 | -0.15 | 0.32 | 0.05 | 0.12 | 0.05 | 0.06 | 0.13 | 0.07 | 1.00 | 0.27 | 0.27 | 0.37 |
| INSULIN_M | 0.14 | 0.26 | 0.25 | -0.39 | 0.22 | 0.43 | 0.56 | 0.37 | -0.28 | -0.19 | 0.31 | -0.36 | 0.29 | 0.18 | 0.32 | 0.37 | 0.34 | 0.34 | 0.15 | 0.27 | 1.00 | 0.98 | 0.42 |
| INSULIN_R | 0.14 | 0.27 | 0.26 | -0.39 | 0.23 | 0.43 | 0.57 | 0.38 | -0.28 | -0.19 | 0.32 | -0.36 | 0.29 | 0.18 | 0.32 | 0.35 | 0.38 | 0.34 | 0.16 | 0.27 | 0.98 | 1.00 | 0.42 |
| LEPTIN | 0.16 | 0.15 | 0.09 | -0.01 | 0.08 | 0.17 | 0.52 | -0.16 | 0.11 | 0.09 | 0.10 | -0.02 | 0.29 | -0.18 | -0.02 | 0.04 | 0.04 | 0.09 | -0.08 | 0.37 | 0.42 | 0.42 | 1.00 |

**Supporting Table S4: Rank correlation matrix. Health 2000, men and women combined.**
