# Supplementary material for: Thirty-One Novel Biomarkers as Predictors for Clinically Incident Diabetes
Source: PLoS One. 2010 Apr 9;5(4):e10100. doi: 10.1371/journal.pone.0010100 (PMC2852424; doi:10.1371/journal.pone.0010100)
Supplement: Table S5 — Gender-specific net reclassification improvement (NRI) due to the biomarker score. Coefficients from FINRISK97 are applied to the Health 2000 validation cohort. (0.06 MB DOC) [file pone.0010100.s006.doc]

| **Predicted risk without biomarker score** | **Predicted risk with biomarker score** | | | | | |
| --- | --- | --- | --- | --- | --- | --- |
| Men^*^ developing diabetes during 7-year follow-up (n=94) | <3% | 3-7.9% | 8-14.9% | ≥ 15% | up^‡^ | down^‡^ |
| <3% | 16 (78.5%) | 4 (21.5%) | 0 (0.0%) | 0 (0.0%) |  |  |
| 3-7.9 % | 5 (14.2%) | 18 (47.7%) | 12 (31.8%) | 2 (6.4%) | 30 (29.4%) | 10 (9.6%) |
| 8-14.9% | 0 (0.0%) | 2 (10.7%) | 6 (32.9%) | 11 (56.5%) |  |  |
| ≥ 15% | 0 (0.0%) | 1 (4.6%) | 1 (4.0%) | 24 (91.4%) |  |  |
|  |  |  |  |  |  |  |
| Men^*^ not developing diabetes during 7-year follow-up (n=2197) | | | | | | |
| <3% | 1223 (91.7%) | 107 (8.0%) | 3 (0.2%) | 1 (0.1%) |  |  |
| 3-7.9 % | 223 (38.9%) | 271 (47.4%) | 63 (11.1%) | 15 (2.6%) | 226 (10.4%) | 348 (16.0%) |
| 8-14.9% | 15 (8.1%) | 76 (40.8%) | 58 (30.9%) | 38 (20.3%) |  |  |
| ≥ 15% | 2 (2.6%) | 12 (15.1%) | 21 (26.8%) | 43 (55.5%) |  |  |
|  |  |  |  |  |  |  |
| Women^†^ developing diabetes during 7-year follow-up (n=80) | | | | | | |
| <3% | 13 (61.5%) | 8 (38.5%) | 0 (0.0%) | 0 (0.0%) |  |  |
| 3-7.9 % | 4 (13.7%) | 22 (69.8%) | 3 (10.0%) | 2 (6.5%) | 20 (23.9%) | 10 (12.5%) |
| 8-14.9% | 1 (5.4%) | 3 (16.1%) | 8 (41.8%) | 7 (36.7%) |  |  |
| ≥ 15% | 0 (0.0%) | 0 (0.0%) | 2 (15.3%) | 12 (84.7%) |  |  |
|  |  |  |  |  |  |  |
| Women^†^ not developing diabetes during 7-year follow-up (n=2694) | | | | | | |
| <3% | 1791 (95.7%) | 78 (4.2%) | 2 (0.1%) | 0 (0.0%) |  |  |
| 3-7.9 % | 166 (29.9%) | 327 (59.1%) | 57 (10.3%) | 4 (0.7%) | 164 (6.3%) | 222 (8.5%) |
| 8-14.9% | 2 (1.6%) | 37 (29.3%) | 64 (50.8%) | 23 (18.3%) |  |  |
| ≥ 15% | 1 (1.5%) | 5 (7.4%) | 11 (16.0%) | 51 (75.2%) |  |  |

**Supporting Table S5: Gender-specific net reclassification improvement (NRI) due to the biomarker score.** Coefficients from FINRISK97 are applied to the Health 2000 validation cohort.

^*^For men, the best biomarker score consisted of a linear combination of adiponectin, apoB, IL-1ra and ferritin. The NRI was 25.4% (SE 0.064), p<0.0001. The conventional risk factor model included the same risk factors as in Fig. 1.

^†^ For women, the best biomarker score consisted of a liner combination of adiponectin, apoB, CRP and insulin. The NRI was 13.6% (SE 0.067), p=0.041. The conventional risk factor model included the same risk factors as in Fig. 1.

^‡^The numbers of persons reclassified up and down do not exactly equal to the sum of different categories, because the reclassification analysis has been performed using the Kaplan-Meier approach [8],[9] and the result has been rounded to the nearest integer.
