# Supplementary material for: Thirty-One Novel Biomarkers as Predictors for Clinically Incident Diabetes
Source: PLoS One. 2010 Apr 9;5(4):e10100. doi: 10.1371/journal.pone.0010100 (PMC2852424; doi:10.1371/journal.pone.0010100)
Supplement: Table S6 — Equations for the best biomarker scores for men, women, and men and women combined (0.02 MB DOC) [file pone.0010100.s007.doc]

| Score_men= -0.074421 x adiponectin + 1.987903 x apoB + 0.162311 x ferritin^1/3^ + 0.079618 x IL-1Ra^1/3^ |
| --- |
| Score_women= -0.078329 x adiponectin + 0.907691 x apoB + 0.438194 x CRP^1/3^ + 0.442380 x insulin^1/3^ |
| Score_combined= -0.081199 x adiponectin + 1.721547 x apoB + 0.154238 x CRP^1/3^ + 0.087740 x ferritin^1/3^ |

**Supporting Table S6: Equations for the best biomarker scores for men, women, and men and women combined**

Beta coefficients have been estimated in FINRISK97 and then applied to the Health 2000 cohort.
