# Supplementary figures and images for: Thirty-One Novel Biomarkers as Predictors for Clinically Incident Diabetes
Source: PLoS One. 2010 Apr 9;5(4):e10100. doi: 10.1371/journal.pone.0010100 (PMC2852424; doi:10.1371/journal.pone.0010100)

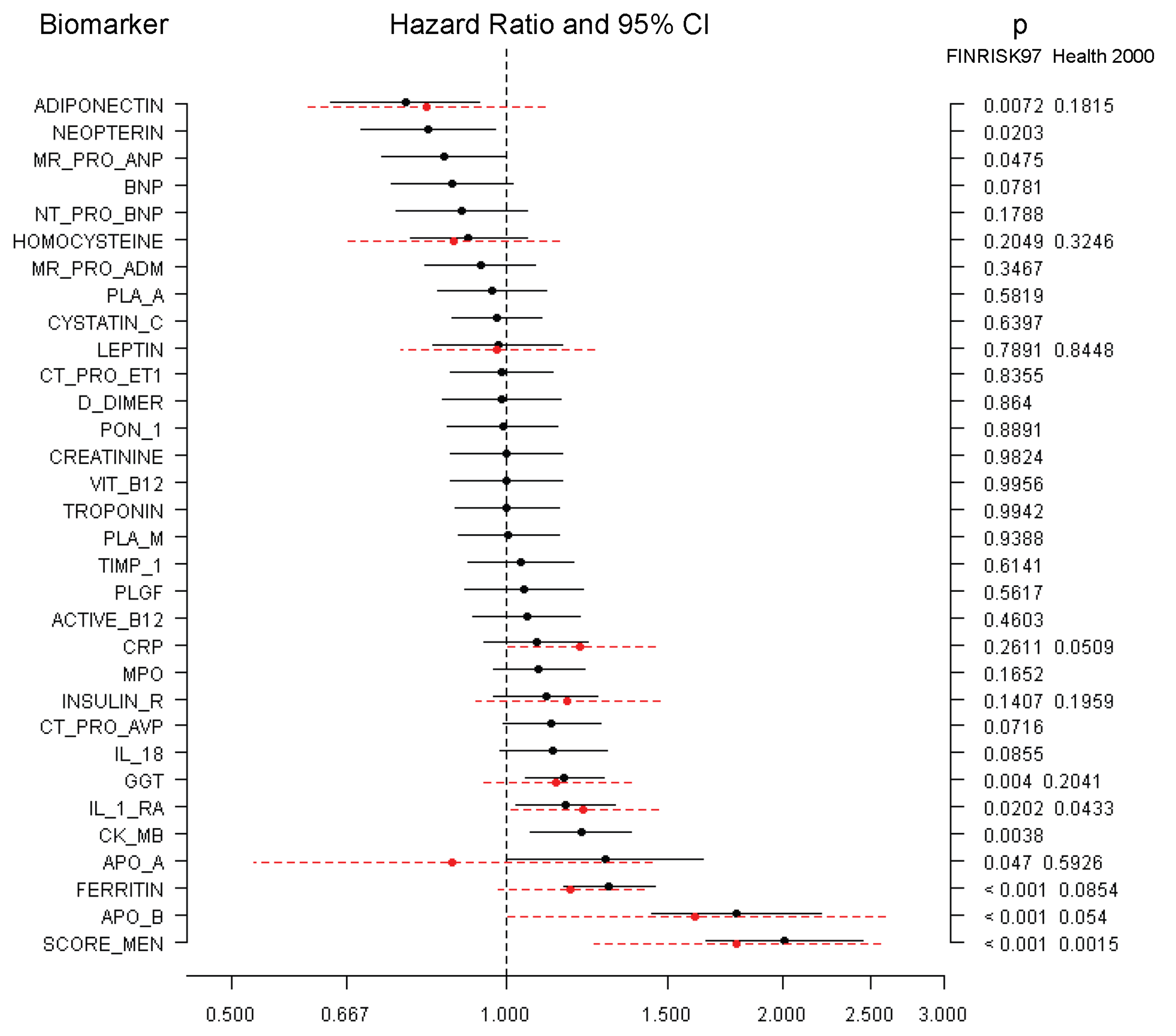

Supplement: Figure S1 — Hazard ratios (95% CI, per one SD) of clinically incident diabetes among men. FINRISK97 cohort (black solid lines) has 249 cases and 3,673 noncases among men. Selected biomarkers and the biomarker score were determined in the Health 2000 cohort (red dotted lines), which has 95 cases and 2,178 noncases. Adjusted for non-HDL-cholesterol, HDL-cholesterol, triglycerides, BMI, systolic blood pressure, current smoking, blood glucose, history of a cardiovascular disease event and use of antihypertensive medication. Age was used as the time scale. The biomarker score for men included adiponectin, apoB, ferritin and IL-1ra. (3.74 MB TIF) [file pone.0010100.s008.tif]

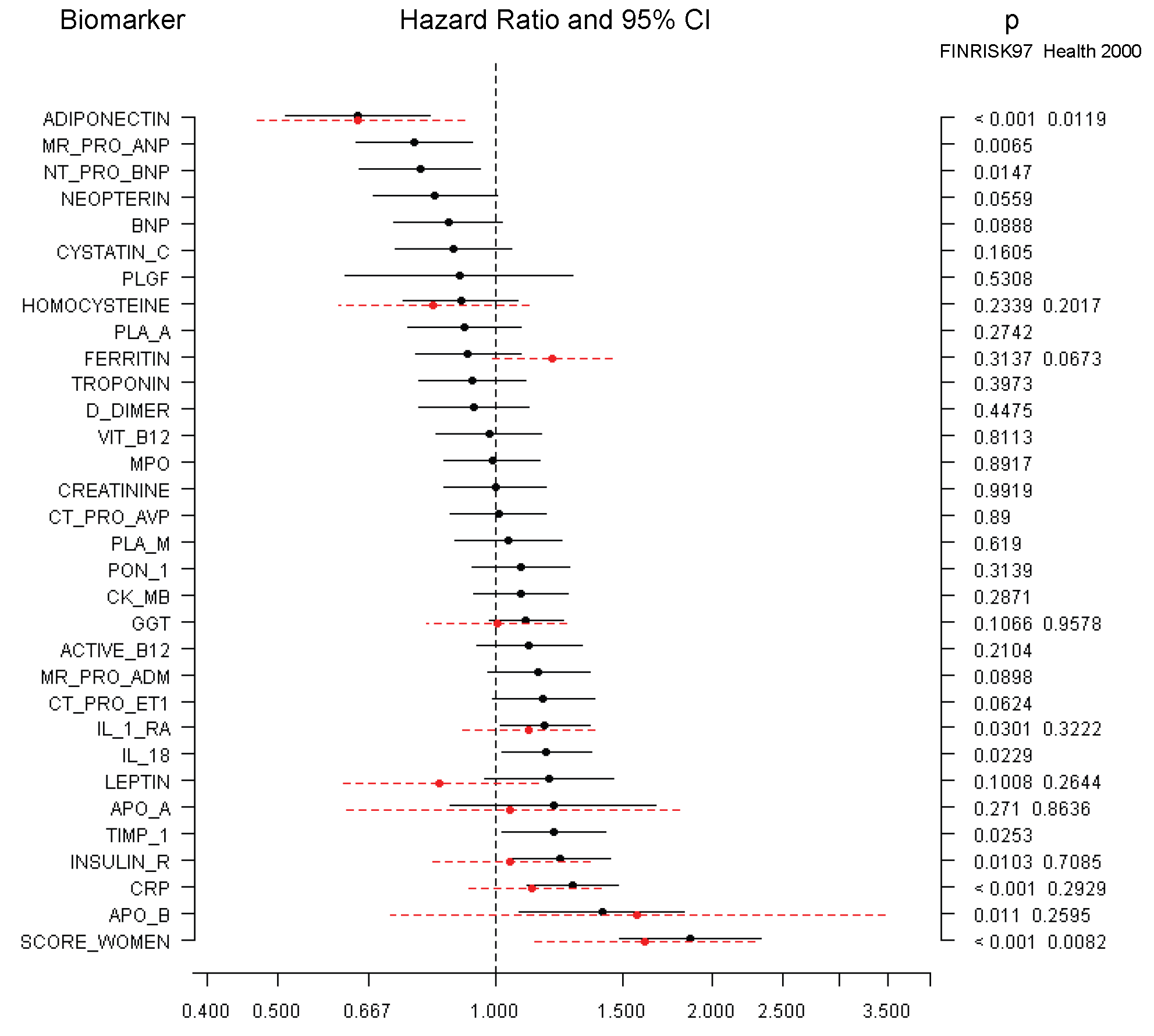

Supplement: Figure S2 — Hazard ratios (95% CI, per one SD) of clinically incident diabetes among women. FINRISK97 cohort (black solid lines) has 168 cases and 3,737 noncases among women. Selected biomarkers and the biomarker score were determined in the Health 2000 cohort (red dotted lines), which has 84 cases and 2,620 noncases. Adjusted for non-HDL-cholesterol, HDL-cholesterol, triglycerides, BMI, systolic blood pressure, current smoking, blood glucose, history of a cardiovascular disease event and use of antihypertensive medication. Age was used as the time scale. The biomarker score for women included adiponectin, apoB, CRP, and insulin. (0.35 MB TIF) [file pone.0010100.s009.tif]
